# Mitigating Cancer Therapy–Related Cognitive Impairment by Targeted Activation of Undruggable Phosphatase

**DOI:** 10.1002/advs.202520135

**Published:** 2026-07-28

**Authors:** Zhimeng Yao, Yuhua Meng, Huanyi Li, Yinhui Jiang, Xiaona Lin, Mengyuan Hu, Qing Liu, Xiaofu Qiu, Hongzheng Ren, Yunlong Pan, Bin Pan, Zexiong Guo, Shuyao Zhang, Dianzheng Zhang, Li Yang, Shegan Gao, Weijing Deng, Jianfan Chen, Hao Zhang

**Affiliations:** ^1^ Department of Urology Surgery The First Affiliated Hospital of Jinan University Jinan University Guangzhou Guangdong China; ^2^ State Key Laboratory of Bioactive Molecules and Druggability Assessment Institute of Precision Cancer Medicine and Pathology School of Medicine Jinan University Guangzhou Guangdong China; ^3^ Postdoctoral Research Station of Basic Medicine Jinan University Guangzhou Guangdong China; ^4^ Department of Pharmacy The First Affiliated Hospital of Jinan University Jinan University Guangzhou Guangdong China; ^5^ Department of Pathology The First People's Hospital of Foshan Foshan Guangdong China; ^6^ Department of Urology Guangdong Second Provincial General Hospital Guangzhou Guangdong China; ^7^ Department of Pathology Gongli Hospital Naval Medical University Shanghai China; ^8^ Department of General Surgery The First Affiliated Hospital of Jinan University Jinan University Guangzhou Guangdong China; ^9^ Department of Pharmacy Jinan University Affiliated Guangzhou Red Cross Hospital Guangzhou Guangdong China; ^10^ Department of Biomedical Sciences Philadelphia College of Osteopathic Medicine Philadelphia Pennsylvania USA; ^11^ School of Life Sciences Guangzhou University Guangzhou Guangdong China; ^12^ College of Clinical Medicine Henan Key Laboratory of Cancer Epigenetics The First Affiliated Hospital of Henan University of Science and Technology Luoyang Henan China; ^13^ Department of Pathology Pudong Gongli Hospital Shanghai University of Medicine & Health Sciences Shanghai China; ^14^ School of Basic Medical Sciences Changzhi Medical College Changzhi Shanxi China; ^15^ MOE Key Laboratory of Tumor Molecular Biology Guangzhou Red Cross Hospital Jinan University Guangzhou Guangdong China; ^16^ Department of Thoracic Surgery The First Affiliated Hospital of Jinan University Guangzhou Guangdong China

**Keywords:** cognitive dysfunction, exosome‐based delivery system, life quality of cancer survivors, saRNA delivery to brain, small RNA activation, targeted therapy for CTRCI, tyrosine phosphatase

## Abstract

Cancer therapy‐related cognitive impairment (CTRCI) is a debilitating neurotoxic condition adversely impacting cancer patients during and post‐cancer treatments. The cancer treatments linked to CTRCI include chemotherapy, hormone therapy, targeted therapy, and immunotherapy. Despite CTRCI severely affecting the psychological and social, cognitive functions, and the overall quality of life of cancer survivors, no effective medications are available currently. Our prior studies have indicated hippocampal tyrosine phosphatase protein tyrosine phosphatase receptor type O (PTPRO) as a putative target for CTRCI. However, phosphatase is historically considered undruggable, and delivering drugs across the blood‐brain barrier (BBB) is challenging. Here, we developed a novel delivery system using neuron‐targeted extracellular vesicles (EVs) engineered with a neuron‐specific peptide rabies virus glycoprotein (RVG) to transport a small activating RNA (saRNA) targeting *Ptpro* (RVG‐EVs‐sa*Ptpro*). We evaluated the stability, dynamic distribution, cytotoxicity, and brain specificity of RVG‐EVs‐sa*Ptpro* in cellular and animal models. A single intravenous injection of RVG‐EVs‐sa*Ptpro* resulted in sustained elevation of PTPRO in the brain for at least 28 days in CTRCI mice. More importantly, RVG‐EVs‐sa*Ptpro* significantly alleviated CTRCI symptoms by enhancing neuronal survival, neurogenesis, and synaptic plasticity. These findings highlight the potential of RVG‐EVs‐sa*Ptpro* system for targeted treatment of CTRCI.

## Graphical abstract

An RVG‑engineered exosomal saRNA delivery system (RVG‑EVs‑sa*Ptpro*) effectively targets and activates hippocampal PTPRO, functioning as a “molecular brake” to alleviate cancer therapy‑related cognitive impairment (CTRCI) by enhancing neuronal survival, neurogenesis, and synaptic plasticity. These findings highlight the potential of the RVG‑EVs‑saRNA system as a targeted therapeutic strategy for CTRCI.

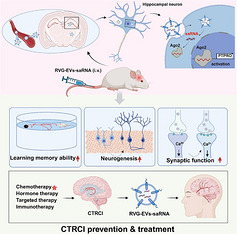

## 1 Introduction

Cancer therapy‐related cognitive impairment (CTRCI) is a debilitating neurotoxic condition adversely impacting cancer patients during and post‐chemotherapy and some other cancer treatments [1, 2, 3, 4, 5]. CTRCI, historically referred to as chemobrain or chemofog, is not only associated with traditional therapies such as chemotherapy and radiation, but also extends to new generations of hormone therapy, targeted therapy, and immunotherapy [5, 6, 7, 8, 9, 10, 11]. On average, approximately 75% of patients undergoing chemotherapy suffer from CTRCI, with older individuals affected more severely [4]. Even though CTRCI impacts treatment outcomes and diminishes the quality of life, there is no effective treatment against CTRCI [4]. Currently available strategies are focused on symptomatic relief, cognitive interventions, physical activity, psychophysical training, and adjunctive medications [6]. However, none of these approaches has been proven efficient and satisfactory, and therefore, more effective treatments for CTRCI are urgently needed.

Recent evidence underscores that neurotoxicity represents a prevalent and clinically significant complication of both conventional and emerging cancer therapies. However, the pathophysiological mechanisms underlying central and peripheral nervous system injury remain largely undefined, and current pharmacological interventions have achieved only modest and variable benefits in clinical settings [1]. In this context, we and others have previously reported that protein tyrosine phosphatase receptor type O (PTPRO), a member of the R3 subfamily of receptor protein tyrosine phosphatases (PTPs), is not only enriched in the hippocampus but also functions critically in synapse formation and neurogenesis in the hippocampus [12, 13, 14]. In addition, the levels of hippocampal PTPRO decline during aging, and reduced levels of PTPRO are highly related to the severity of CTRCI [12]. Our previous findings demonstrated that *Ptpro* deletion increases doxorubicin (DOX)‐induced CTRCI in both healthy and tumor‐bearing mice [12]. We have also found that restoring the levels of PTPRO in a region‐specific manner can alleviate DOX‐induced CTRCI in *Ptpro* ^−/−^ mice [12], suggesting that restoring hippocampal PTPRO is a promising approach in preventing and/or treating CTRCI. Mechanistically, PTPRO‐mediated suppression of SRC and EPHA4 phosphorylation/activation likely protects against neurodegeneration and enhances neurogenesis in the CTRCI mouse model [12]. However, PTPs were historically considered “undruggable” due to their highly conserved active site, the phosphotyrosine (pTyr)‐binding pocket [15, 16, 17]. Additionally, PTPRO's role as a tumor suppressor complicates drug development.

RNA‐based therapeutics have presented a revolutionary potential to transform how we treat diseases [18]. Small activating RNAs (saRNAs), double‐stranded RNAs with 20–26 nucleotides, can upregulate gene expression transcriptionally in an Ago2‐dependent manner [19]. saRNA targeting tumor suppressors like CEBPA, P21, and TP53 have entered clinical and preclinical trials [19, 20, 21, 22, 23, 24]. We have previously engineered a saRNA delivery system with antibody‐conjugated nanoparticles, which is capable of not only restoring the level of PTPRO in breast cancer cells but also overcoming their trastuzumab resistance [25]. Although several targeted delivery systems (e.g., nanoliposomes, exosomes) have been reported to successfully deliver various RNA molecules—including siRNA, miRNA, circRNA, and modified self‐amplifying RNA—to the brain, targeted delivery of small activating RNA to the brain remains unachieved [24, 26].

Extracellular vesicles (EVs), nature's nanoparticles with low immunogenicity, unique bio‐distribution capabilities, and BBB‐crossing potential, are ideal drug carriers [27, 28, 29, 30, 31, 32, 33]. Rabies virus glycoprotein (RVG) peptides‐modified EVs have delivered siRNAs to neurons in neurological disease models [34, 35]. In the present study, we engineered RVG‐EVs to deliver saRNA targeting *Ptpro* to the brain. Our results demonstrate that RVG‐EVs‐sa*Ptpro* effectively restores hippocampal PTPRO and alleviates CTRCI symptoms in the animal model, offering a novel delivery system and targeted therapeutic strategy for CTRCI and other neurological diseases.

## 2 Results

### 2.1 Preparation and Characterization of RVG‐EVs‐saRNA

**FIGURE 1:**
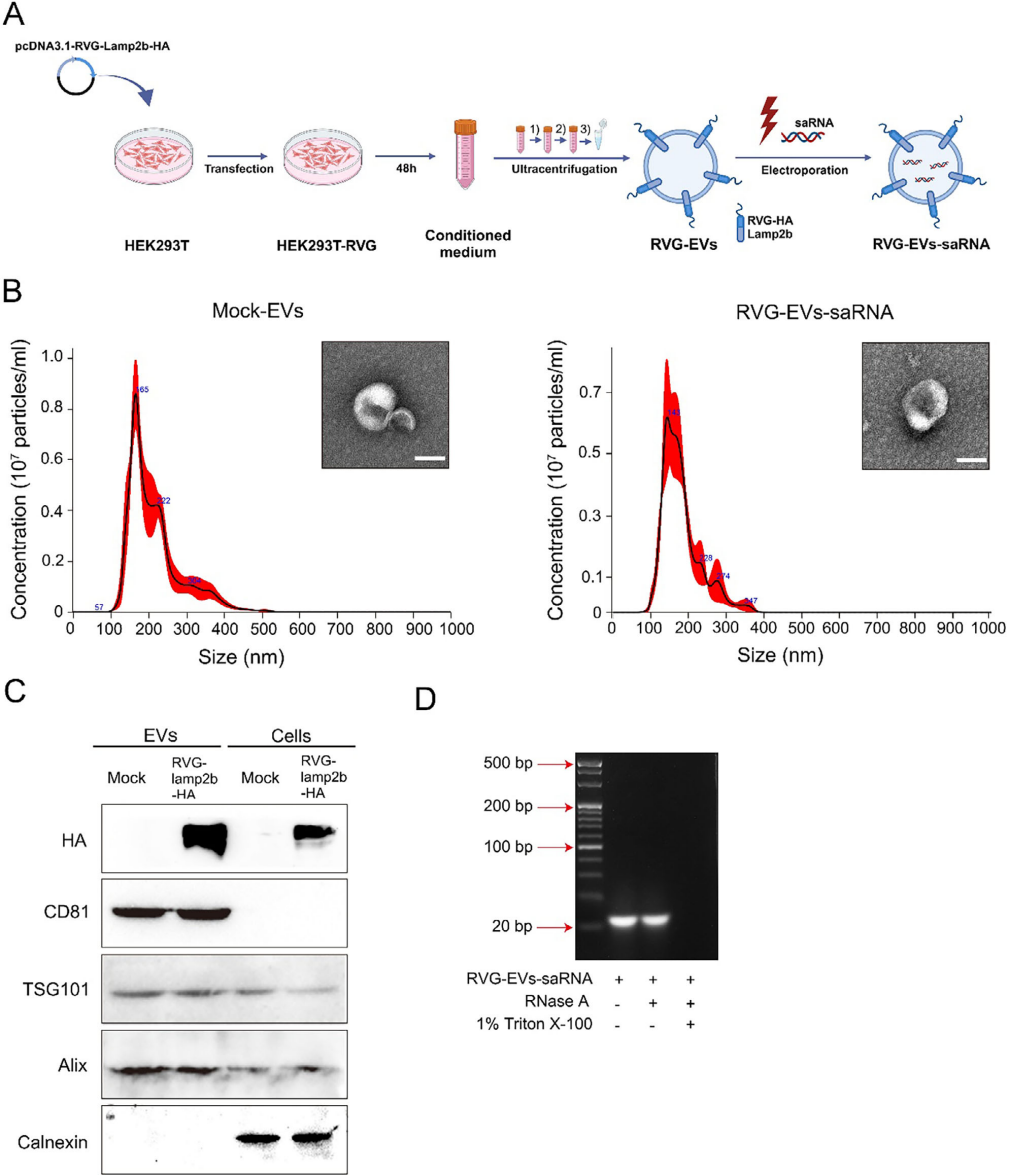
Preparation and characterization of engineered RVG‐EVs. (A) Schematic diagram of the production and harvest of engineered RVG‐EVs for targeted PTPRO up‐regulation via small activating RNA delivery. Ultracentrifugation: (1) 500 × g 10 min; (2) 10 000 × g 30 min; (3) 100 000 × g 60 min. (B) NTA and TEM of mock EVs and RVG‐EVs‐saRNA isolated from the culture medium of HEK293T cells. Scale bar: 100 nm. (C) Western blot analysis of HA, CD81, TSG101, ALIX, and Calnexin from EVs prepared from supernatants of 293T that had been transfected with RVG‐Lamp2b‐HA or mock treated. (D) Agarose gel electrophoretic analysis of saRNA in RVG‐EVs after treatments with RNase A/T1 Mix and 1% Triton X‐100 for 30 min.

In this study, we constructed an exosomal drug delivery system for brain‐targeted delivery of the small activating RNA that targets murine *Ptpro* (sa*Ptpro*) for CTRCI treatment. We transfected the plasmid encoding RVG‐Lamp2B‐HA into human embryonic kidney (HEK) 293T cells. The RVG‐EVs were purified from the culture supernatants by ultracentrifuge and loaded with saRNA by electroporation (Figure 1A). We first designed five saRNAs according to the design protocol [36] and verified that sa*Ptpro*‐158 significantly activated PTPRO in mouse hippocampal cells using Western blot and RT‐qPCR analysis (Figure S1A–C). Meanwhile, electroporation at 400 V and 125 µF under the 100 µL sample volume yielded the greatest retention of electroporated material, and this condition was used for subsequent experiments (Figure S2A,B). The physicochemical properties of EVs were characterized by transmission electron microscopy (TEM) and nanoparticle tracking analysis (NTA). The size peaks (in diameter) of mock EVs and RVG‐EVs‐saRNA were determined by NTA to be 169 ± 15.9 nm and 151 ± 5.5 nm (mean ± SEM), respectively (Figure 1B). Immunoblotting of HA‐tagged RVG and exosome‐specific markers (CD81, TSG101, ALIX, and Calnexin) indicated that the pellets isolated from cultured cells were mainly RVG‐EVs (Figure 1C). We then evaluated the stability of saRNA encapsulated in RVG‐EVs after RNase treatment. Agarose gel electrophoretic analysis demonstrated that free saRNA was degraded by RNases while the RNA band was intact when the saRNA was encapsulated in RVG‐EVs (Figure 1D). This indicated that EV encapsulation could protect the saRNA from RNase degradation. Taken together, these data confirm that RVG‐EVs‐saRNA was successfully produced as designed.

### 2.2 Engineered RVG‐EVs Were Efficiently Internalized by Neurons and Delivered saRNA to Activate *Ptpro* Expression

**FIGURE 2:**
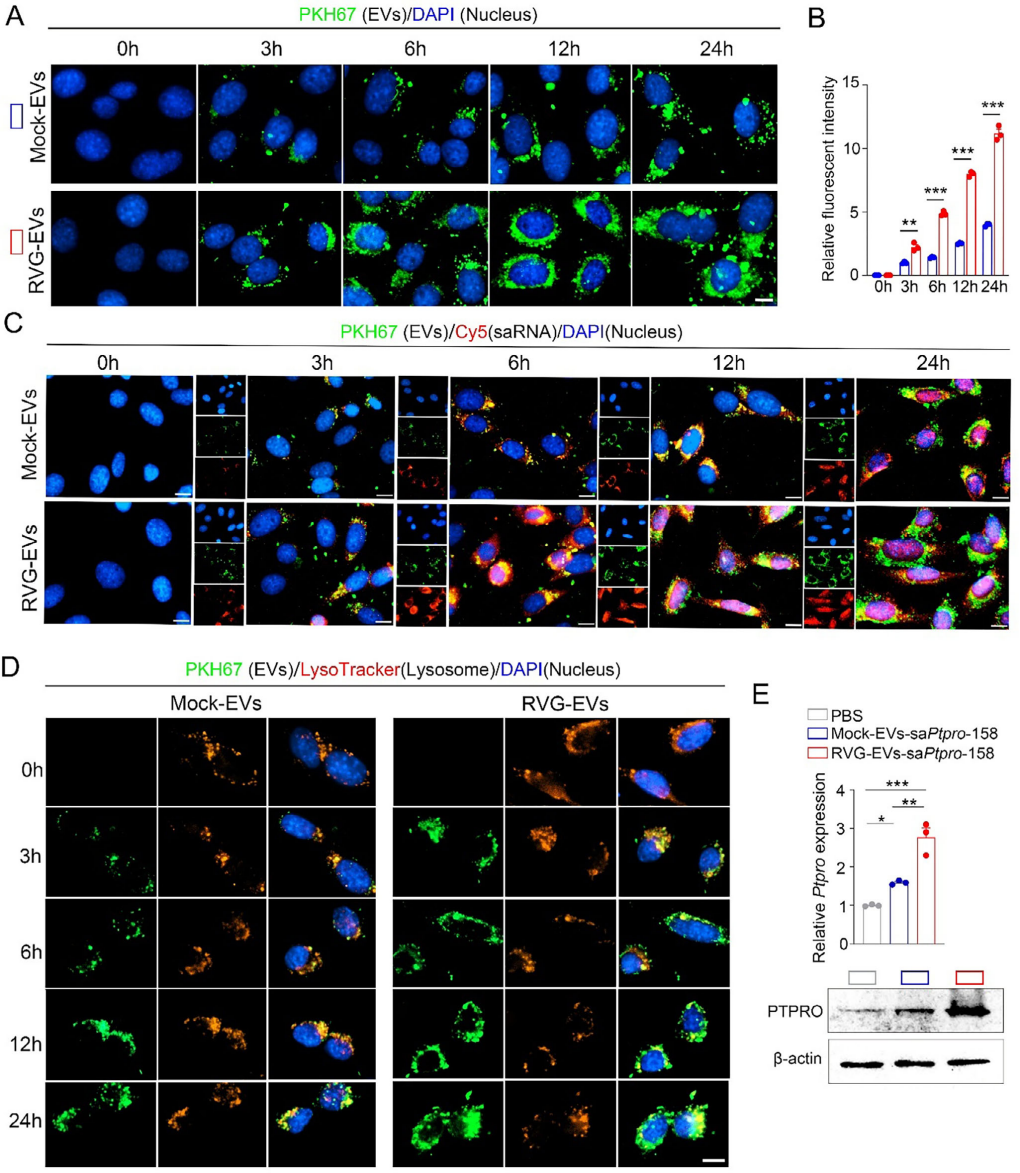
Internalization and biological effects of RVG‐engineered EVs in HT‐22 cells. (A) Fluorescence images depicting the ratio of EVs uptake by HT‐22 cells after co‐culturing with 100 µg PKH67‐labeled mock‐EVs or RVG‐EVs (PKH67, green) at 0, 3, 6, 12, and 24 h. Scale bar: 10 µm. Nuclei are stained blue (DAPI). (B) Quantitative fluorescence analysis of PKH67 in HT‐22 cells after incubation with 200 µg PKH67‐labeled mock EVs or RVG‐EVs. (C) Colocalization of saRNA and mock‐EVs and RVG‐EVs in HT‐22 cells after incubation for 0, 3, 6, 12, and 24 h. PKH67‐labeled EVs are represented in green, and Cy5‐saRNA is displayed in red. Scale bar: 20 µm. (D) Lysosomal escape of mock‐EVs and RVG‐EVs in HT‐22 cells was assessed after incubation for 0, 3, 6, 12, and 24 h, with lysosomes labeled using Lyso Tracker Red. Scale bar: 10 µm. (E) PTPRO expression in HT‐22 cells after 48 h (upper panel, RT‐qPCR) or 72 h (bottom panel, immunoblotting) treatment with PBS, mock‐EVs, or RVG‐EVs. Using Student's *t* test (B) and one‐way ANOVA (E) to analyze statistical differences. n.s., not significant; ^***^ *p* < 0.001.

Efficient cellular uptake is a critical step for RNA‐based drugs. We first evaluated the uptake of 100 µg mock‐EVs (untargeted) or RVG‐EVs (neuron‐targeting) in neurons after different durations of incubation by fluorescence microscopy. The EVs were labeled with the membrane fluorescent dye PKH67. As co‐incubation time increased, the fluorescence intensity of PKH67‐labeled mock EVs and RVG‐EVs in HT‐22 cells (a mouse hippocampal neuronal cell line) gradually increased (Figure 2A). The fluorescence intensity and aggregation rate of RVG‐EVs were significantly higher than those of mock‐EVs (Figure 2B). Next, we further investigated the cellular uptake and intracellular trafficking behavior of RVG‐EV‐encapsulated saRNA after different durations of incubation (0, 3, 6, 12, and 24 h) in vitro. As shown in Figure 2C, both the fluorescence intensity of Cy5‐labeled saRNA and PKH67‐labeled RVG‐EVs were increased in HT‐22 cells by extending the incubation time. The fluorescence intensity of the Cy5‐saRNA was aggregated in the cytoplasm and showed strong co‐localization with PKH67‐labeled RVG‐EVs at 3 h after incubation, indicating that RVG‐EVs‐saRNA could be taken up by neurons and further validating the successful design of an EVs‐based saRNA delivery system (Figure 2C). Six hours after incubation, we only observed a minor fluorescence co‐localization between Cy5‐saRNA and PKH67‐labeled RVG‐EVs in the cytoplasm, and a minor population of cells had a strong nuclear fluorescence intensity of the Cy5‐saRNA. The fluorescence signals of Cy5‐saRNA were evenly dispersed all over the cytoplasm without aggregation at this time point, indicating that the saRNA had escaped from the lysosomes into the cytoplasm (Figure 2C). After 24 h, there was minimal fluorescence co‐localization between Cy5‐saRNA and PKH67‐labeled RVG‐EVs, and the majority of cells showed a strong nuclear fluorescence intensity of the Cy5‐saRNA, indicating that most of the saRNA had entered the nucleus, the cellular compartment where it would carry out its intended function (Figure 2C). In addition, efficient lysosomal escape and subsequent cytoplasmic distribution are essential prerequisites for saRNA‐based therapeutics to exert their biological activity. As shown in Figure 2D, both mock‐EVs and RVG‐EVs were internalized into the cells and entrapped within the lysosomes 3 h after incubation, evidenced by a large area of co‐localization of the EVs signal (green) and the Lyso Tracker signal (red) showing as bright yellow fluorescence. At the 6‐h time point, mock‐EVs continued to exhibit strong lysosomal co‐localization, suggesting limited lysosomal escape efficiency. On the contrary, the RVG‐EVs illustrated a remarkable swift dispersion in the cytoplasm, demonstrating enhanced neuronal internalization and more efficient intracellular trafficking. Importantly, prolonged incubation resulted in progressive lysosomal escape of RVG‐EVs, with increasing fluorescence signal distributed throughout the neuronal cytoplasm. This observation demonstrates RVG‐EVs’ ability to bypass the conventional endocytosis‐endosome pathway, enabling direct cytoplasmic delivery of encapsulated saRNA. In contrast, mock‐EVs persistently accumulated in endosomal compartments following cellular uptake. Furthermore, we investigated the capability of RVG‐EVs‐sa*Ptpro*‐158 to activate *Ptpro* expression in HT‐22 cells. Compared with mock‐EVs‐sa*Ptpro*‐158‐treated cells, RVG‐EVs‐sa*Ptpro*‐158 treatment for 72 h was able to effectively activate *Ptpro* expression in HT‐22 cells, as shown by RT‐qPCR, and increase PTPRO protein levels, as shown by immunoblotting (Figure 2E). This increase in PTPRO could be attributed to the enhanced cellular uptake and the efficient translocation of sa*Ptpro*‐158 into the nucleus. These findings suggest that RVG‐tagged exosomes encapsulating sa*Ptpro*‐158 are efficiently internalized by neurons, transported to the nucleus, and ultimately upregulate the PTPRO protein level in the neurons.

### 2.3 Engineered RVG‐EVs Enabled Efficient Targeted Delivery of saRNA to the Brain

**FIGURE 3:**
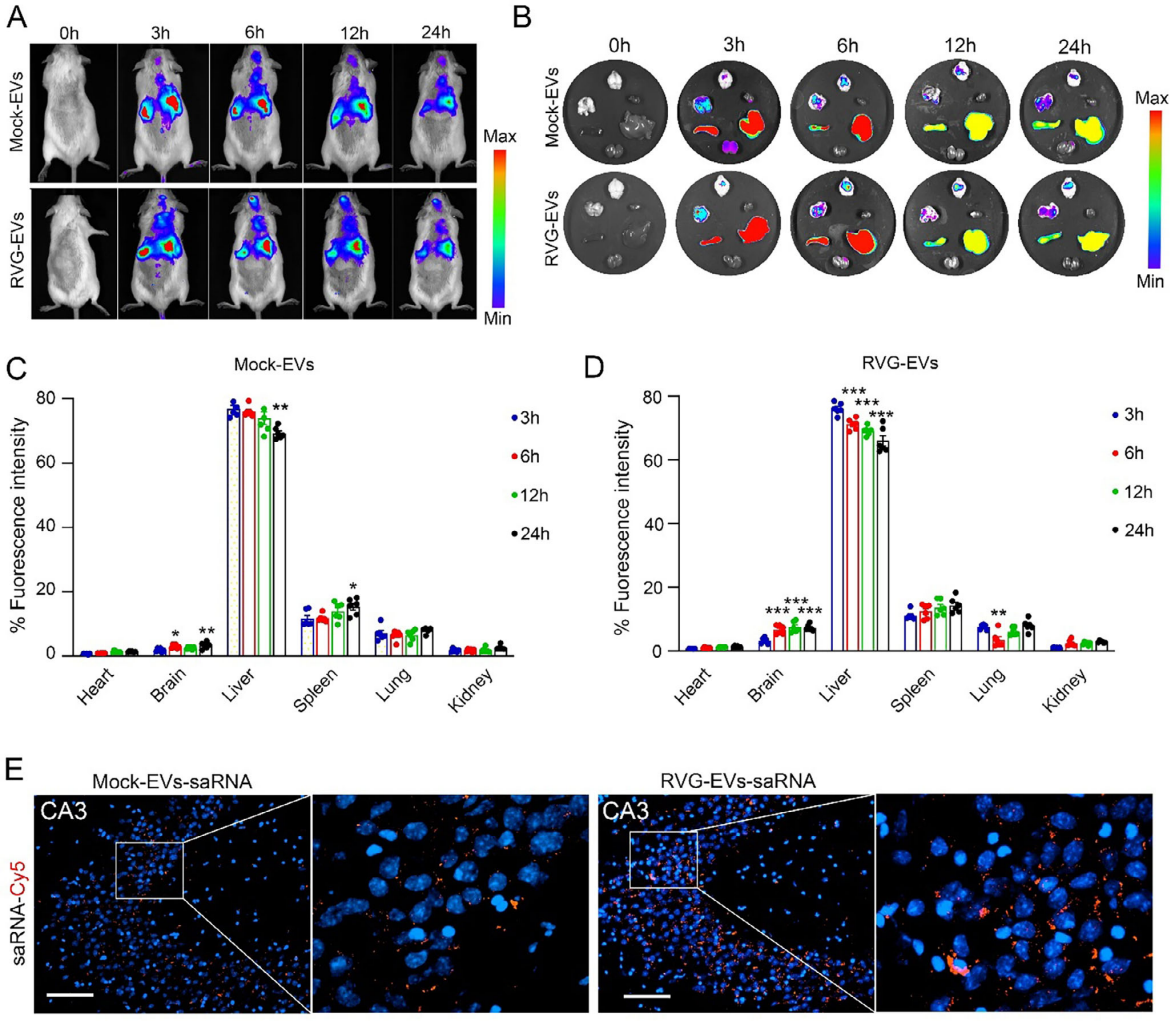
Biodistribution of RVG‐engineered EVs encapsulated with saRNA in vivo. (A) Representative live fluorescence distribution imaging of the mice at 3, 6, 12, and 24 h after intravenous administration of 150 µg DiR‐labeled mock EVs or RVG‐EVs, respectively. (B) Individual organs (i.e., liver, spleen, lung, heart, kidney, and brain) were isolated from normal mice after intravenous administration of DiR‐labeled mock EVs or RVG‐EVs at 0, 3, 6, 12, and 24 h and bioimaged using the IVIS imaging system. (C‐D) Quantitation of fluorescence intensities in different organs of normal mice after intravenous injection of DiR‐labeled mock EVs (C) or RVG‐EVs (D) at the indicated time points. ^*^ *p* < 0.05, ^**^ *p* < 0.01, ^***^ *p* < 0.001 vs. the 3 h group using one‐way ANOVA followed by the Holm‐Sidak post hoc multiple comparison test. (E) Distribution of Cy5‐saRNA in the mouse hippocampal CA3 region at 24 h post‐injection with mock‐EVs or RVG‐EVs encapsulated with Cy5‐saRNA through the tail vein. Scale bar: 250 µm.

Having determined the uptake and function of RVG‐EVs in vitro, we next investigated the biodistribution of RVG‐EVs in vivo. We used the near‐infrared fluorescent cyanine dye, DiR, to label lipid bilayer membranes for near‐infrared in vivo imaging. DiR‐labeled mock‐EVs or RVG‐EVs were injected via the tail vein of healthy mice, and then the intensity and distribution of near‐infrared fluorescence were evaluated using an in vivo imaging system (IVIS) at several time points between 3 and 24 h post‐injection (Figure 3A). In line with previous studies, both mock‐EVs and RVG‐EVs initially distributed rapidly to organs of the mononuclear phagocyte system (also known as the reticuloendothelial system, RES), with the highest accumulation in the liver, followed by the spleen and the lungs [37, 38, 39]. At 24 h post‐injection, there was still intense activity in the liver and spleen (Figure 3A). At 6 h after injection, we observed that the highest accumulation of RVG‐EVs in the brain occurred and decreased slightly with time, which was due to being intercepted and captured by the RES (Figure 3A). The fluorescence intensity of DiR‐labeled RVG‐EVs in brain regions was significantly higher than that of mock EVs at several periods of time, confirming RVG‐engineered EVs could effectively target the brain (Figure 3A). Moreover, due to signal interference in vivo, we further harvested the major organs (brain, heart, liver, spleen, lung, and kidney) at different time points after DiR‐labeled mock‐EVs or RVG‐EVs injection to record and quantify the fluorescence intensity ex vivo to accurately identify from which tissue the signal originated (Figure 3B). Based on the quantification of the fluorescence intensities in different organs, most EVs were found in peripheral organs 24 h post injection (Figure 3C,D). Specifically, fluorescence intensity in the liver was the highest among all organs within 3 h post‐administration (mock‐EVs, 77% ± 2.4; RVG‐EVs, 76% ± 1.8). Thereafter, a sharp decrease in fluorescence intensity was observed over time in peripheral organs, most notably the liver (mock‐EVs, 77% ± 2.4% to 69% ± 1.8, *p* < 0.001; RVG‐EVs, 76% ± 1.8% to 66% ± 3.9, *p* < 0.001). This rapid clearance is attributable to the RES, wherein accumulated exosomes are efficiently taken up and degraded by resident macrophages. Upon bypassing peripheral clearance and entering the brain, RVG‐EVs exhibited a slower decline over time compared to that observed in peripheral organs. Then, by fluorescence analysis of individual organs from mice after injection with DiR‐labeled mock‐EVs or RVG‐EVs for 24 h, we observed that the mouse brains treated with RVG‐EVs had higher levels of fluorescence than the mouse brains treated with mock‐EVs (Figure S3A). Organs of the RES (i.e., the liver, the spleen, and the lungs) showed high fluorescence signals. Other organs (i.e., the heart and the kidneys) only showed weak fluorescence signals (Figure S3A). In addition, the co‐localization of Alexa Fluor 488‐labeled anti‐HA antibody (binding to HA‐tagged RVG/Lamp2B fusion protein on the EVs membrane, green signal) and the DiR‐labeled EVs (red signal) was observed in the hippocampus of the mouse after RVG‐EVs injection, and no co‐localization fluorescence signals were detected in the hippocampus of the mock‐EVs‐treated mouse, suggesting that the DiR‐labeled EVs are reliable for tracking EVs in vivo (Figure S3B). These results indicated that the engineered RVG‐EVs could more efficiently penetrate across the BBB into the brain in vivo. Furthermore, the brain was excised 24 h after EVs administration, and frozen sections were made to evaluate the distribution of Cy5‐sa*Ptpro* in the hippocampus. As expected, the hippocampus CA3 region of mice treated with RVG‐EVs‐sa*Ptpro*‐158 had stronger fluorescence intensity of Cy5‐labeled sa*Ptpro*‐158 than that of mock‐EVs‐sa*Ptpro*‐158‐treated mice (Figure 3E).

Next, we continued to explore the duration of saRNA action in the hippocampi of mice after a single intravenous administration of 150 µg RVG‐EVs‐sa*Ptpro*‐158 or mock‐EVs‐sa*Ptpro*‐158. We observed that the *Ptpro* activation in the hippocampi of mice treated with a single intravenous administration of RVG‐EVs‐sa*Ptpro*‐158 persisted for at least 28 days, while little or no *Ptpro* activation was observed in the hippocampi of mice 7 days after treatment with the same dose of mock‐EVs‐sa*Ptpro*‐158 (Figure S4A–C). Notably, administration of either RVG‐EVs‐sa*Ptpro*‐158 or mock‐EVs‐sa*Ptpro*‐158 showed no detectable effects on major organs, indicating that the EVs treatment was well‐tolerated in mice without observable adverse effects (Figure S4D). These results demonstrated that RVG‐EVs were able to deliver sa*Ptpro*‐158 efficiently into the hippocampi of the brain and upregulate *Ptpro* gene expression for almost one month in mice.

### 2.4 Therapeutic Efficacy of RVG‐EVs‐sa*Ptpro*‐158 in Aged CTRCI Mice

**FIGURE 4:**
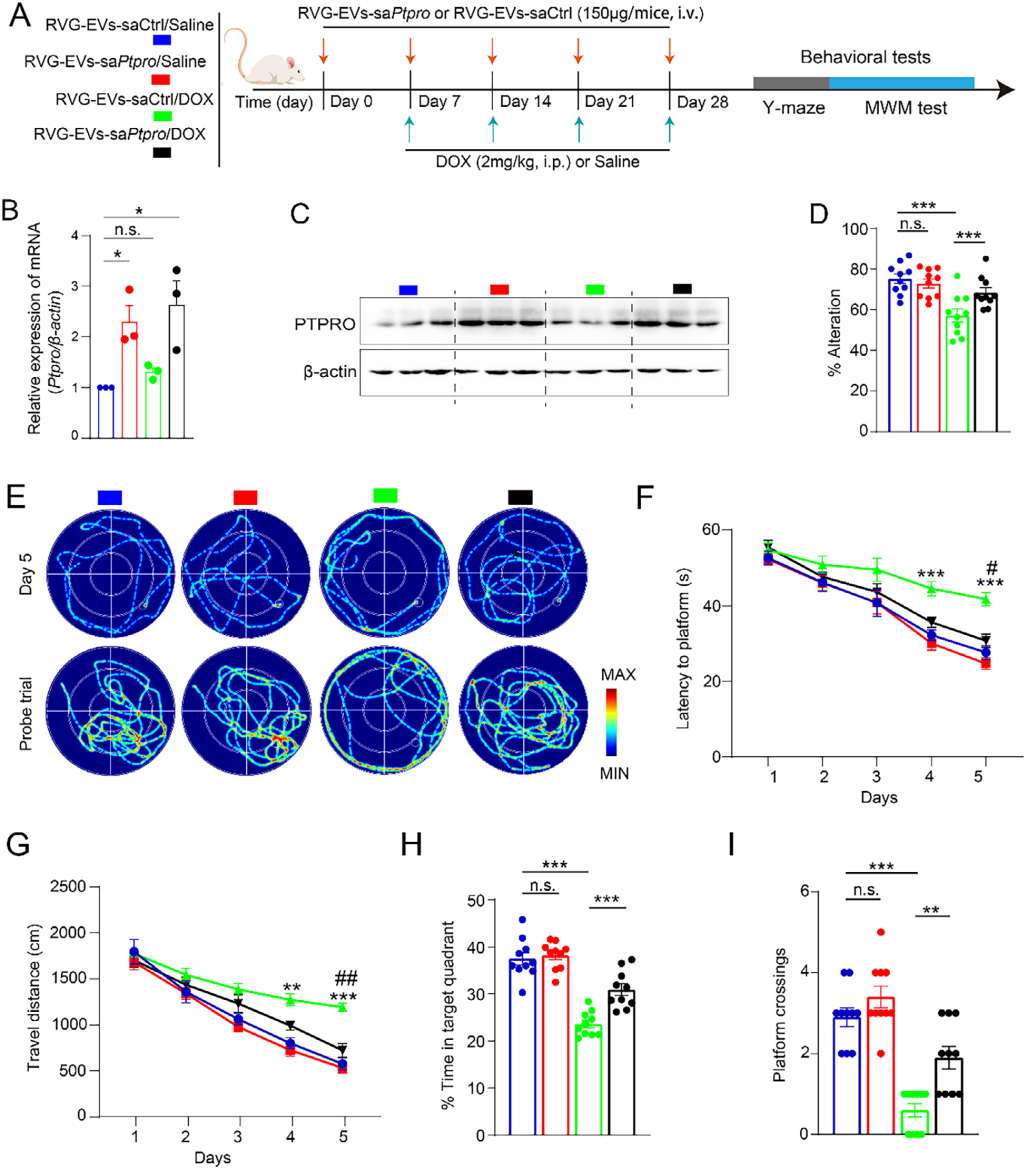
Delivery of saRNA by RVG‐EVs caused *Ptpro* up‐regulation and alleviated DOX‐induced CTRCI in aged mice. (A) Schematic of the experimental design. (B, C) The expression levels of PTPRO in the hippocampi of mice under various treatments were analyzed by qRT‐PCR (B) and immunoblotting (C). (D) The changes in spontaneous alternation during the 10 min test sessions in the Y‐maze test. *n* = 10 per group. (E) Representative swimming traces plot in the MWM test. *n* = 10 per group. (F) The time to reach the submerged platform. (G) The distances traveled before reaching the submerged platform. (H) The time spent in the target quadrant. (I) The number of crossings before reaching the target location. Error bars: SEM. n.s., not significant; ^*^ *p* < 0.05, ^**^ *p* < 0.01, ^***^ *p* < 0.001; ^#^ *p* < 0.05, ^##^ *p* < 0.01 by two‐way ANOVA (B, D, H, and I) or three‐way ANOVA (F, G) followed by a Tukey‐Kramer post hoc test.

**FIGURE 5:**
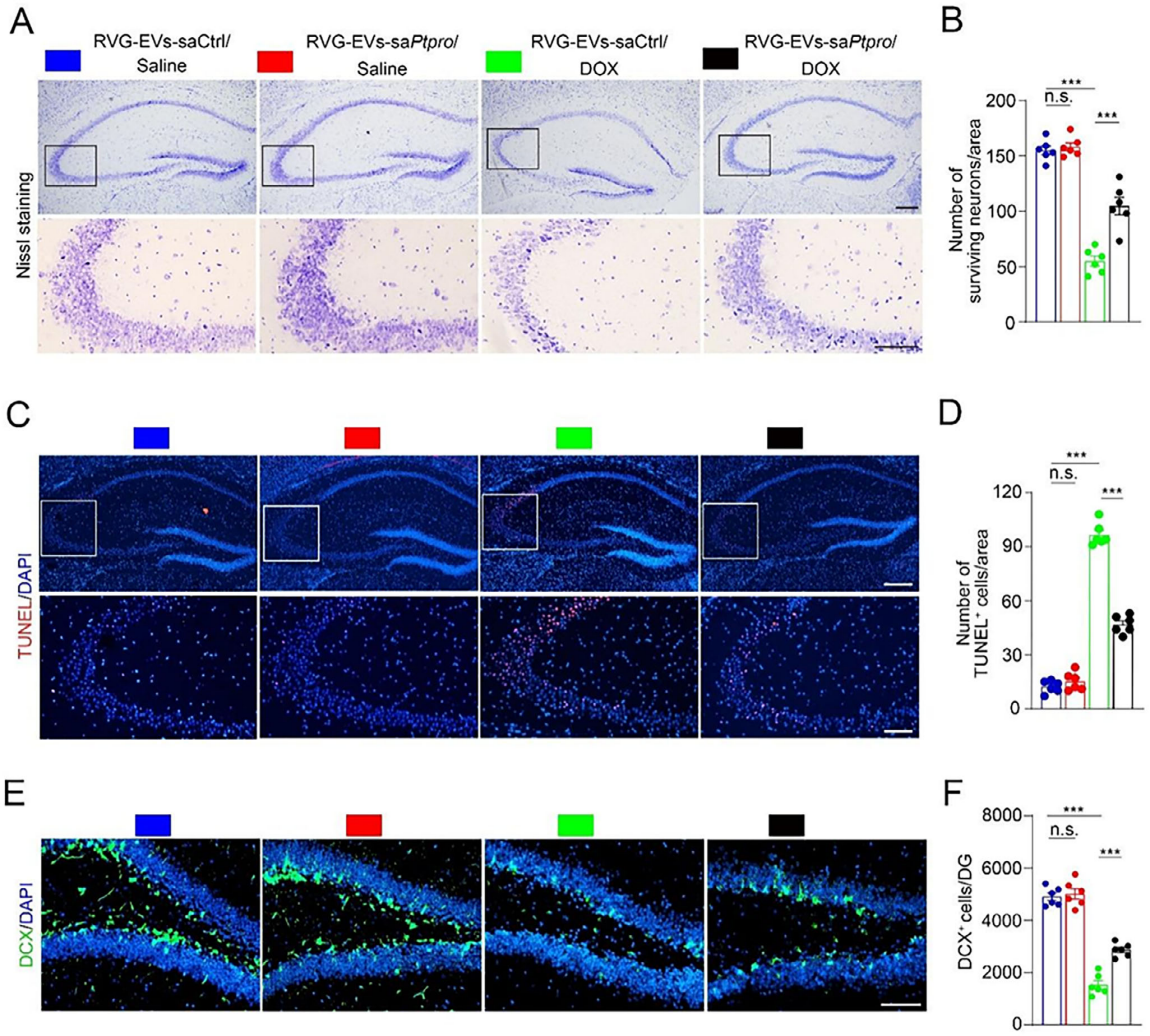
RVG‐EVs‐mediated delivery of sa*Ptpro* prevents neurodegeneration and promotes neurogenesis in aged mice treated with DOX. (A) Nissl staining of the hippocampi (upper panel) and hippocampal CA3 region (bottom panel) of mice under various treatments. Scale bars: 200 µm (upper panel), 100 µm (bottom panel). (B) Quantification of surviving neurons in the hippocampal CA3 region of mice. *n* = 6 per group. (C) TUNEL staining of the hippocampi (upper panel) and hippocampal CA3 region (bottom panel) of mice under various treatments. Scale bars: 250 µm (upper panel), 100 µm (bottom panel). (D) Quantification of TUNEL‐positive neurons in the hippocampal CA3 region of mice. *n* = 6 per group. (E) Representative images of immature (DCX^+^) neurons in the hippocampi. Scale bars: 100 µm. (F) Quantification of immature neurons in the hippocampi. *n* = 6 per genotype. DG, dentate gyrus. Error bars: SEM. n.s., not significant; ^***^ *p* < 0.001 by one‐way ANOVA followed by a Tukey‐Kramer post hoc test.

**FIGURE 6:**
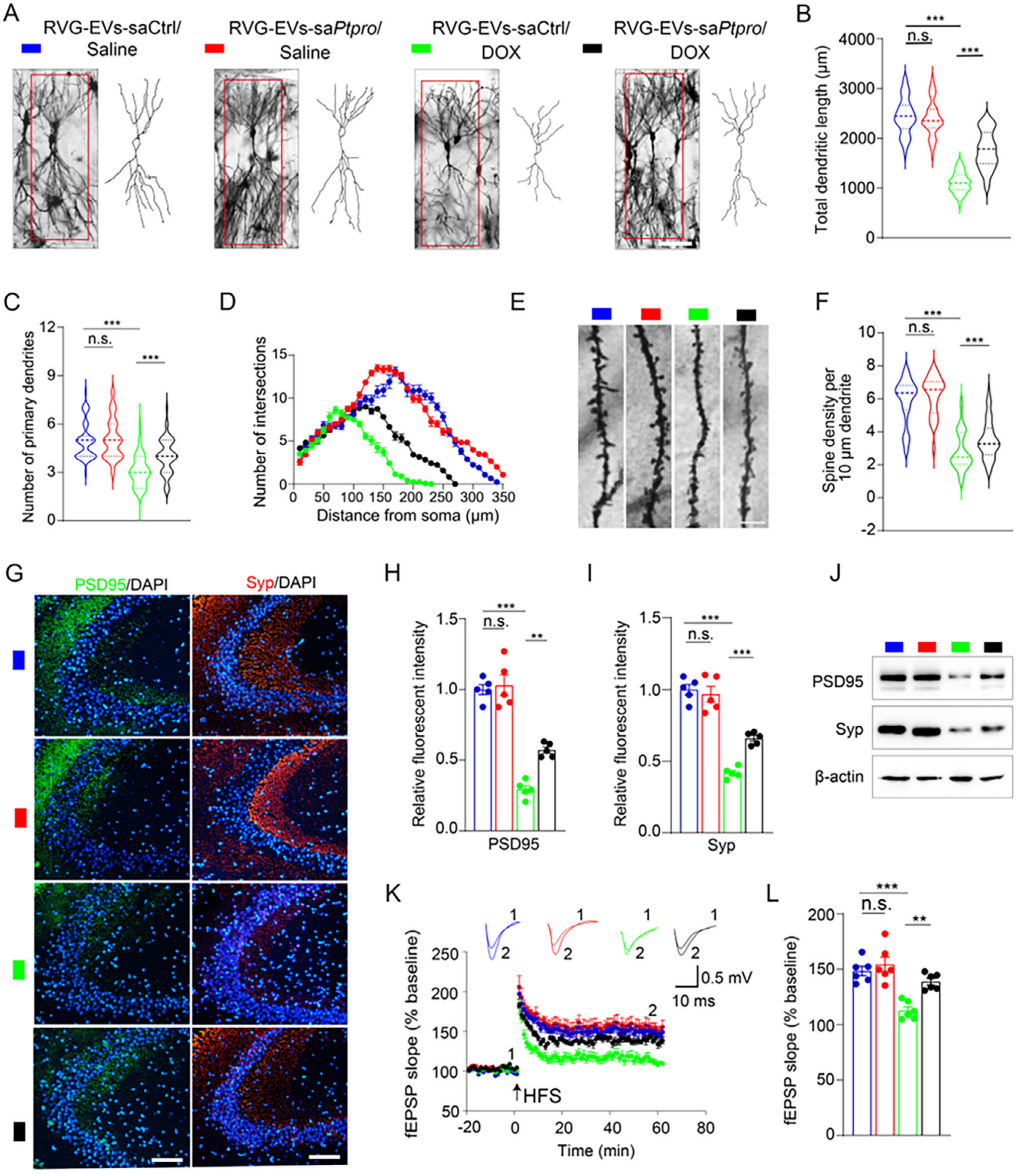
RVG‐EVs‐mediated delivery of sa*Ptpro* ameliorates synaptic function in aged mice treated with DOX. (A) Representative Golgi‐Cox staining of hippocampal CA3 pyramidal neurons in each group. Scale bar: 50 µm. (B,C) Quantification of the total dendritic length (B) and primary dendrites (C) of CA3 pyramidal neurons. *n* = 4 per group. (D) Sholl's analysis of the complexity of CA3 pyramidal neurons. *n* = 4 per group. (E) Representative photomicroscopy images of Golgi‐stained dendrites of CA3 pyramidal neurons. Scale bars: 5 µm. (F) Quantitative analysis of spine densities in CA3 pyramidal neurons. *n* = 6 per group. (G) Representative immunofluorescence images of Syp and PSD95 in hippocampal CA3 sections. Scale bars: 200 µm. (H) Quantification analysis of the average fluorescence intensity of PSD95 in hippocampal CA3 sections. *n* = 5 per group. (I) Quantification analysis of the average fluorescence intensity of Syp in hippocampal CA3 sections. *n* = 5 per group. (J) Immunoblotting of Syp and PSD95 in the hippocampi of mice. *n* = 3 per group. (K) The time course of fEPSP measurements was recorded in CA3 by tetanic stimulation (100 Hz for 1 s) applied to the mossy fiber. (L) The averaged fEPSPs were recorded 56–60 min after the induction of LTP. *n* = 6 slices from 4−6 mice. Error bars: SEM. n.s., not significant; ^**^ *p* < 0.01, ^***^ *p* < 0.001 by one‐way ANOVA followed by a Tukey‐Kramer post hoc test (B, C, F, H, I, and L).

To date, CTRCI is highly prevalent (up to 75%) in women with breast cancer and is associated with poor outcomes and quality of life. It's been confirmed that female sex is a higher risk for CTRCI [40, 41]. The sex‐specific vulnerability to CTRCI has significant implications for understanding of the mechanisms underlying the occurrence of CTRCI [40, 41]. Therefore, we give priority to female mice to evaluate the therapeutic potential of RVG‐EVs‐sa*Ptpro*‐158 in a CTRCI mouse model. Given that CTRCI is particularly prevalent in elderly cancer patient populations, our preclinical protocol utilized aged mice (18 months old) that were pretreated with RVG‐EVs‐sa*Ptpro*‐158 (or saline), followed by treatment with DOX (or saline) once a week for 4 consecutive weeks (Figure 4A), and followed by behavioral tests including the Y‐maze and the MWM tests. After the end of the treatment course, the mRNA and protein levels of PTPRO in the hippocampus of mice were estimated by RT‐qPCR and immunoblotting assays, respectively. Hippocampal *Ptpro* expression in mice treated with RVG‐EVs‐sa*Ptpro*‐158 was significantly upregulated at the mRNA (Figure 4B) and protein levels (Figure 4C) compared with those of RVG‐EVs‐saCtrl/saline or RVG‐EVs‐saCtrl/DOX treatment groups, and DOX exposure had no effect on *Ptpro* expression. Our previous studies have found that age‐related decline in hippocampal PTPRO contributed to CTRCI‐related decline in cognition, and synaptic plasticity. To test whether activation of hippocampal PTPRO could reverse DOX‐induced cognitive impairment, we conducted the Y maze and the MWM tests to evaluate the hippocampus‐associated cognitive function in terms of short‐term spatial memory and learning ability of the different treatment groups. As expected, RVG‐EVs‐sa*Ptpro*‐158 administration effectively reduced DOX‐induced cognitive behavioral dysfunctions (Figure 4D–I). Whereas performance in the first two days was governed mainly by non‐learning factors, including stress and anxiety, with minimal intergroup variance, robust learning effects emerged around Day 4 after several days of training and memory consolidation. Our findings collectively demonstrate that RVG‐EVs‐sa*Ptpro*‐158 treatment effectively improves hippocampus‐dependent spatial learning and memory in mice (Figure 4F,G). In addition, RVG‐EVs‐sa*Ptpro*‐158 treatment showed no effect on cognitive performance, as evidenced by equivalent Y‐maze and MWM results between RVG‐EVs‐saCtrl/saline and RVG‐EVs‐sa*Ptpro*/saline groups, confirming its safety profile in non‐DOX‐exposed mice (Figure 4D–I). Since PTPRO is highly expressed in the CA3 region (Figure S5), localizes to the postsynaptic membrane of excitatory neurons and functions as a potent initiator of synapse formation [13]. Next, we performed Nissl staining to conduct morphometric analyses of hippocampal CA3 neurons across treatment groups, further evaluating the neuroprotective efficacy of RVG‐EVs‐sa*Ptpro* against DOX‐induced chemotherapy‐related cognitive impairment (CRCI). Nissl staining demonstrated that RVG‐EVs‐sa*Ptpro*‐158 treatment significantly attenuated DOX‐induced neuronal deterioration and reduced neuronal loss in the CA3 region (Figure 5A,B). Consistent with these findings, TUNEL staining revealed that RVG‐EVs‐sa*Ptpro*‐158 administration markedly decreased DOX‐triggered apoptotic cell death in the CA3 hippocampus (Figure 5C,D). Moreover, RVG‐EVs‐sa*Ptpro*‐158 treatment rescued DOX‐impaired neurogenesis, as evidenced by the preservation of DCX‐labeled neuronal progenitor cells in the subgranular zone (SGZ) of the dentate gyrus (Figure 5E,F). These data suggest that RVG‐EVs‐mediated *Ptpro* activation prevented DOX from inducing neuronal apoptosis and impairing neurogenesis in the hippocampus, consistent with our previous report that PTPRO played a role in preventing neurodegeneration and promoting neurogenesis [12]. In addition, RVG‐EVs‐sa*Ptpro*‐158 administration ameliorated synaptic dysfunction in the hippocampus of mice treated with DOX by effectively rescuing DOX‐induced decreases in total dendritic length, number of primary dendrites, dendritic complexity, and dendritic spine density of CA3 pyramidal neurons (Figure 6A–F). DOX exposure revealed reduced synaptophysin (gene name: *Syp*, an essential presynaptic vesicle membrane protein) and postsynaptic density protein‐95 (PSD95, gene name: *Dlg4*, a postsynaptic scaffold protein) levels in area CA3 of the hippocampus in mice compared to controls, which were rescued by RVG‐EVs‐sa*Ptpro*‐158 treatment (Figure 6G–J). Next, we assessed hippocampal synaptic plasticity by performing long‐term potentiation (LTP) recordings at mossy fiber synapses. Consistently, RVG‐EVs‐sa*Ptpro*‐158 administration significantly ameliorated DOX‐induced learning and memory impairment, which was accompanied by rescuing impaired hippocampal mossy fiber‐CA3 synaptic activity (Figure 6K,L). Collectively, these results demonstrate that PTPRO activation via RVG‐EV delivery represents a promising therapeutic strategy to prevent CTRCI, particularly in aging populations.

## 3 Discussion

Cancer survivors frequently face persistent and debilitating treatment‐related sequelae, among which CTRCI has emerged as a particularly challenging complication with limited therapeutic options [1, 2, 6]. The convergence of two age‐associated conditions—cancer and cognitive decline—has rendered CTRCI a growing public health concern with substantial socioeconomic consequences [4]. Current strategies, including drug repurposing (e.g., pioglitazone) and natural product investigation (e.g., berberine alkaloids, omega‐3 fatty acids, and various polyphenols), have demonstrated limited clinical efficacy for CTRCI management [42]. In this study, we demonstrated that RVG‐EVs‐saRNA‐mediated PTPRO expression significantly ameliorated CTRCI‐induced symptoms in aged mice. Given its demonstrated BBB permeability, excellent safety profile, and high tolerability, the engineered RVG‐EV‐mediated saRNA delivery system represents a promising therapeutic platform for both prevention and treatment of CTRCI.

Given that the mechanisms underlying CTRCI remain poorly defined and represent a major limitation in developing effective interventions, we sought to explore the molecular determinants contributing to neural protection and recovery [1, 2, 12]. We have previously reported that (1) hippocampal PTPRO is capable of protecting against DOX‐induced CTRCI, and (2) age‐related decline of brain PTPRO is one of the driving forces in CTRCI development and progression [12]. Together with findings showing that saRNA‐induced hippocampal restoration of PTPRO effectively alleviated CTRCI symptoms in aged mice [12]. These findings indicate that PTPRO is a potent therapeutic target for CTRCI treatment. Beyond its well‐characterized roles in neural development [12, 13, 14]. Results from genome‐wide association studies (GWAS) uncovered a close association between the level of PTPRO and cognitive function [43]. PTPRO also plays important roles in the pathogenesis of Parkinson's disease (PD) [44, 45]. PTPRO is thus likely involved in both physiological and pathological development of neurons and cognition. Therefore, PTPRO could potentially serve as a therapeutic target for both CTRCI and other cognitive and neurological diseases.

Targeting PTPRO pharmacologically has been challenging. First, PTPRO is a member of the protein tyrosine phosphatases (PTPs) family, which have thus far been considered “undruggable” primarily due to their highly conserved active enzymatic sites [15, 16, 17, 25, 46, 47, 48]. Second, we and others have also found that PTPRO is expressed and acts as a putative tumor suppressor in a variety of cancer types [25, 47, 49, 50, 51, 52, 53]. Successful restoration of hippocampal PTPRO could be used for both CTRCI treatment and cancer prevention. By exploiting AGO2 and components of the RNA‐interacting transcriptional activating complex (RISC), saRNA induced a long‐lasting and sequence‐specific effect to activate or upregulate transcription targets [19, 21]. Compared to other gene‐activation approaches such as CRISPER/Cas9 [54], saRNA is more advantageous [55], because it restores target proteins without changing genetic sequences, thus avoiding the risk of genetic mutation [24]. Several saRNA‐based therapies targeting tumor suppressor genes, such as CEBPA, P21, E‐cad, and TP53, have undergone clinical and preclinical trials [19, 20, 21, 22, 23]. Of note, saRNA therapies in preclinical studies and clinical trials are mainly applied in liver disorders and cancers in bladder, lung, colorectal, breast, pancreas, and prostate (see below discussions) [24, 25]. But due to poor uptake, low stability, and the barrier of BBB, saRNA‐based application has not been reported in neurological diseases [34]. To our knowledge, we are the first to report that saRNA‐mediated therapy can be effectively applied in treating nervous disease.

EVs show therapeutic promise due to their disease‐associated dysregulation and drug delivery potential, making it essential to understand their cellular uptake mechanisms [56, 57]. Cells appear to take up EVs by a variety of endocytic pathways, including endocytosis, micropinocytosis, phagocytosis, and membrane fusion. Indeed, it seems likely that a heterogeneous population of EVs may gain entry into a cell via more than one route. Most experimental evidence identifies endocytosis as the principal pathway for EVs uptake, with resultant accumulation in endosomal compartments [56]. EVs internalization occurs as quickly as 15 min post‐delivery [56]. Endo‐lysosomal sequestration of EVs represents a significant intracellular barrier that substantially compromises the therapeutic efficacy of encapsulated nucleic acids [56, 57]. In our study, we explored the cellular uptake and intracellular trafficking of EVs in cultured cells and in mice. As expected, compared to mock‐EVs, the uptake of RVG‐EVs is significantly elevated in neurons. After an incubation period of 24 h, RVG surface modification of EVs prevents endosomal entrapment and boosts saRNA delivery efficiency. Quantitative analysis revealed that RVG‐EVs‐sa*Ptpro*‐158 increased PTRPO mRNA levels and protein expression compared to controls in neurons. RVG‐EVs effectively crossed the BBB to reach the hippocampus, exhibited high saRNA loading efficiency, and served as an effective delivery system for CTRCI therapy. Therefore, our findings demonstrate that RVG‐EVs delivery system promotes receptor‐mediated transcytosis and thus precision delivery of saRNA.

Hitherto, EV‐based delivery of saRNA remains an untapped frontier. Being a double‐stranded nucleic acid with the nucleobases paired inside and phosphate groups on the outside, saRNA is not favored to interact with the negatively charged cell membrane. In addition, the naked saRNA is rapid capture by the RES (predominantly liver Kupffer cells and splenic macrophages), with subsequent elimination via renal filtration and urinary excretion [58]. Therefore, different carriers, including lipid nanoparticles, dendrimers, lipids, polymer hybrids, and aptamers, have been tried for either local (intratumoral, intravesical, and rectal) or systemic administration with minimal success [24, 59, 60, 61, 62]. Based on the results that saRNA delivered by antibody‐conjugated nanoparticles to the breast cancer cells is capable of restoring PTPRO to overcome trastuzumab resistance [19, 25], we hypothesized that the EVs‐mediated delivery system would be more beneficial due to its safety, biocompatibility, low immunogenicity, unique biodistribution, cargo protection, and enhanced cellular uptake [29, 30, 31, 32, 33]. Although EVs have the inherent ability to cross the biological membrane, such as BBB, only ∼0.5% of unmodified EVs were found in the brain when delivered systemically [58]. To overcome this barrier, we engineered EVs with the brain‐targeting peptide RVG [35], which is accumulated in the brain significantly better than the unmodified EVs. Given the altered distribution pattern at 48 h and potential risks of nonspecific staining and dye accumulation [58], we harvested organs at 24 h post‐intravenous injection. Although the biodistribution of both RVG‐labeled EVs and mock‐EVs principally accumulation in off‐target organs, particularly in the liver after 24 h post‐injection, the RVG‐labeled EVs increased brain uptake by about 2.5‐fold compared to mock‐EVs. Our results corroborate established findings that native EVs exhibit preferential biodistribution to the liver, splenic, and pulmonary systems, with signal persistence observed throughout the 24 h monitoring period [58]. In addition, we also observed that hippocampal PTPRO activation continued for at least 28 days in the group treated with 150 µg of RVG‐EVs‐saRNA via intravenous injection, compared to the mock‐EVs‐saRNA treatment group, suggesting a much longer retention time. Thus, similar to the RVG‐EVs‐delivered siRNA to suppress BACE1 in Alzheimer's models without systemic immunogenicity, the RVG‐EVs‐delivered saRNA should also have minimal off‐target effects [35, 58].

The saRNA‐based treatments can upregulate specific genes with potential applications in clinical therapeutics and safety profiles [25]. This approach has begun to emerge in the field of central nervous system cognitive disorders, offering broader application prospects. While drug repurposing strategies such as pioglitazone offer the advantage of low upfront cost and established safety databases, their clinical efficacy for CTRCI remains unproven, and their adverse effect profiles raise significant concerns for long‐term use in cancer survivors [42, 63, 64]. In contrast, our RVG‐EVs‐saRNA approach represents a mechanistically distinct, disease‐modifying strategy with targeted brain delivery, sustained effect, and a favorable preliminary safety profile.

In conclusion, we have developed an innovative delivery system and demonstrated that the engineered EVs‐delivered saRNA is capable of not only restoring the hippocampal PTPRO but also alleviating the CTRCI symptoms in the animal model by enhancing neuronal survival, neurogenesis, and synaptic plasticity. While RVG decoration enhances brain EVs delivery, the modest 2.5‐fold increase indicates that BBB targeting efficiency remains limited. In the following study, a dual‐targeting strategy can be employed by combining RVG with other peptides (e.g., Angiopep‐2 for blood‐brain barrier penetration) to enhance brain‐targeting specificity. To reduce clearance and immunogenicity, a combined engineering strategy for RVG‐EVs could be developed, involving knockout of immunogenic surface proteins (e.g., MHC‐I) or incorporation of CD47 ‘don't eat me’ signals. In addition, in most studies examining the toxicity profile of EVs, intravenous injection of EVs was safe [34]. For example, repeated chronic injections of HEK293 EVs in mice for more than 22 days produced few deviations in body weight, serum cytokines, and blood cell composition. No systemic effects were observed in mice following intravenous administration of heterologous plant‐ or bovine milk‐derived EVs. Ongoing human clinical trials, including multiple completed first‐in‐human studies, further substantiate the safety of intravenous EVs administration [65]. In summary, future studies should systematically explore loading efficiencies, dose optimization, long‐term safety, and scalability to translate this platform into clinical applications for CTRCI and other neurological disorders.

## 4 Materials and Methods

### 4.1 Cell Culture

HT‐22 (RRID: CVCL_0321) and HEK293T (RRID: CVCL_0063) cell lines were obtained from the National Collection of Authenticated Cell Cultures (Shanghai, China) and cultured in a humidified atmosphere containing 5% CO_2_ at 37°C using the indicated culture medium supplemented with 10% FBS, 100 units/mL penicillin, and 100 units/mL streptomycin, unless indicated otherwise. Murine hippocampal neuronal cell lines HT22 and HEK293T were cultured in DMEM supplemented with 10% FBS. Before EVs isolation, the culture medium of HEK293T cells was replaced by DMEM with 10% of EVs‐depleted FBS and cultured for 48 h, after which the conditioned culture medium was collected for subsequent EVs isolation.

### 4.2 Generation of Stably Transfected Cell Lines

HEK293T cells were seeded onto 10 cm dishes and grown to 80% confluence; the pcDNA3.1‐RVG10‐Lamp2b‐HA (MIAOLING BIOLOGY) with corresponding control vectors were transfected into HEK293T cells using Lipofectamine 3000 (Invitrogen, Carlsbad, CA, USA) according to the manufacturer's instructions. Forty‐eight hours after transfection, hygromycin B (HY‐B0490, MCE, USA) was added to the cell culture medium for another 10 days to obtain stable strains. Resistant colonies were pooled and subcultured in the selection medium.

### 4.3 Preparation and Purification of EVs

HEK293T cells transduced with pcDNA3.1‐RVG10‐lamp2b‐HA were cultured for 48 h in DMEM supplemented with 10% EVs‐depleted FBS; then 293T‐RVG or 293T‐mock were collected from the respective conditioned culture medium by sequential centrifugation (first at 500 × g for 10 min, followed by 10 000 × g for 30 min; EVs‐containing supernatants were filtered through a 0.22‐µm filter (Millipore); then EVs in the filtrate were pelleted by centrifugation at 100 000 × g for 1 h and resuspended in PBS). The Bradford assay (Sangon Biotech, USA) quantified the total protein concentration of EVs preparations.

### 4.4 NTA

Total particles in 293T cell exosome samples were analyzed by nanoparticle tracking (A & P Instrument Co. Ltd., Guangzhou, China) using a NanoSight LM10 system (NanoSight Ltd., Amesbury, UK). Briefly, as described previously, isolated exosome samples were appropriately diluted using PBS buffer and analyzed three times. Data was collected and analyzed using the NTA software (version: NTA 3.4 Build 3.4.003). All measurements were conducted at room temperature.

### 4.5 TEM

Exosome morphological observation was performed using a TEM (JEM‐1400, Hitachi, Shiga, Japan). In brief, exosome particles obtained by ultracentrifugation were suspended in 100 µL PBS, and a 10 µL aliquot of the suspension was loaded onto formvar carbon‐coated grids and incubated for 5 min at room temperature. Next, the exosomes were fixed in 2% paraformaldehyde for 5 min at room temperature and washed three times with PBS. Excess liquid was drained by gently touching the edge of the grid with a piece of clean filter paper. Next, the grid was dipped in 2% uranyl acetate for 1 min and embedded in a mixture of uranyl acetate (0.8%) and methylcellulose (0.13%). Excess solution was wiped off, samples were dried completely, and examined under a TEM.

### 4.6 Characterization of EVs by Western Blotting

EVs were lysed in RIPA buffer (Santa Cruz Biotechnology). Total protein lysates were prepared and analyzed by immunoblotting using anti‐ALIX (Cat. No. 2171; Cell Signaling Technology, Beverly, MA, USA), anti‐TSG101 (Cat. ab133586; Abcam, Cambridge, UK), anti‐CD81 (Cat. ab109201; Abcam, Cambridge, UK), anti‐Calnexin (Cat. ab133615; Abcam, Cambridge, UK), and anti‐HA (Cat. H3663, Sigma, St. Louis, MO, USA) as described previously.

### 4.7 EVs Encapsulation of saRNA by Electroporation

The saRNAs were designed according to previously detailed general design rules [36]. All saRNA sequences were chemically synthesized by TSINGKE, Inc. All saRNA sequences are listed as follows: sa*Ptpro*‐158 forward: 5’‐CCUCGAUCUAUUCAUGCAA[dT][dT]‐3’ and reverse: 5’‐UUGCAUGAAUAGAUCGAGG[dT][dT]‐3’; sa*Ptpro*‐524 forward: 5’‐ CUCGAUCUAUUCAUGCAAA[dT][dT]‐3’ and reverse: 5’‐ UUUGCAUGAAUAGAUCGAG[dT][dT]‐3’; sa*Ptpro*‐525 forward: 5’‐ GUAGCUUUGGUGCCAGCAA[dT][dT]‐3’ and reverse: 5’‐ UUGCUGGCACCAAAGCUAC[dT][dT]‐3’; sa*Ptpro*‐602 forward: 5’‐ CGUUGCUUGUGAUUCUAAA[dT][dT]‐3’ and reverse: 5’‐ UUUAGAAUCACAAGCAACG[dT][dT]‐3’. Other studies were consulted in order to optimise and compare the electroporation parameters [35, 66]. Electroporation was performed in 0.2‐cm cuvettes with aluminum electrodes (Bio‐Rad Laboratories) using a Bio‐Rad Gene Pulser I with a capacitance extender set at 400 V and 100 µF. For every electroporation, the sample volume was fixed at 100 µL, containing 50 µg of EVs and 50 µg of Cy5‐labeled saRNA (TSINGKE, China). After electroporation, the electroporated EVs‐saRNA mixture was treated with RNase to eliminate saRNAs that may be bound to the membrane of exosomes and ultracentrifugated at 100 000 × g for 70 min at 4°C to remove free saRNAs.

### 4.8 EVs Staining

The PKH67 green fluorescent cell ligation kit (NoninBio) was used to label the lipid bilayer of exosomes. Add PKH67 (4 µL), separate exosomes into 1 mL of diluent C, and incubate at room temperature for 5 min; use exosomes without PKH67 staining as a negative control. Bovine serum albumin (1 mL/5%) (Solarbio) was added to stop staining. The PKH67‐labeled EVs mixture was then centrifuged at 100 000 × g for 30 min at 4°C. Wash with PBS and centrifuge at 100 000 × g for 30 min to obtain pure PKH67‐labeled exosomes. The supernatant was removed very carefully, and the pure PKH67‐labeled exosomes were suspended in 100 µL of PBS. The mixture was filtered through a 0.22‐µm filter (Millipore), resuspended in a complete medium, and incubated with HT‐22 cells at 37°C for 0, 3, 6, and 12 h.

### 4.9 Quantitative Real‐Time PCR (RT‐qPCR)

Total RNA was extracted from the cells using the NcmSpin Cell/Tissue Total RNA Kit (NCM Biotech Cat. No. M5105). Complementary DNA synthesis was performed using a BcaBest RNA PCR kit from Takara Bio, Inc. according to the manufacturer's protocols. qPCR was performed using an iQTM5 Multicolor Real‐Time PCR Detection System (Bio‐Rad Laboratories, Inc.) with Realtime PCR Master Mix (SYBR Green, Toyobo Life Science). The thermocycling conditions used for qPCR were as follows: 95°C for 30 s, followed by 40 cycles at 95°C for 5 s and 60°C for 30 s, in a total volume of 20 µL. Relative mRNA expression levels were assessed using the 2‐ΔΔCq method. β‐actin was used as the exogenous control. The PCR primer sequences used were as follows: *Ptpro* Fw: 5’‐TGGCTGCCAGGAATGTGTTA‐3’; Rev: 5’‐TAAGGGGCAGTTCTGTGCTG‐3’; β‐actin Fw: 5’‐TGCACCACCAACTGCTTAGC‐3’; Rev: 5’‐GGCATGGACTGTGGTCATGAG‐3’.

### 4.10 Agarose Gel Electrophoretic Analysis

Agarose was dissolved at 4% in 0.1 m His/0.1 m MES buffer (pH 6.1) and heated for 3 min to allow uniform dissolution. The gels were stained with GS‐GelRed nucleic acid gel dye (GL802; Genesand) and then poured into a flatbed in which a comb was inserted. After the addition of the sample, electrophoresis was carried out at a constant voltage of 120 V for 40 min at room temperature. After electrophoresis, the gels were exposed to iBrightFL1000. For each condition, the experiment was run at least twice.

### 4.11 RVG‐EVs Uptake Imaging

HT‐22 cells were seeded in 6‐well plates at a density of 50–60 × 10^4^ cells/well. After 24 h of culture, 100 µg mock‐EVs or RVG‐EVs loaded with saRNA stained with PKH67 were incubated with cells for 0, 3, 6, 12, and 24 h. The cell culture medium was discarded and washed three times with PBS. Cells were fixed with 4% PFA fixative for 20 min at room temperature and in the dark, and the formaldehyde was removed by washing with PBS three times. Subsequently, nuclear staining and photography were performed.

### 4.12 Lysosomal Escape

To confirm lysosomal escape of RVG‐EVs, HT‐22 cells were incubated with 100 µg of PKH67‐labeled mock‐EVs or RVG‐EVs for 0, 3, 6, 12, or 24 h. Cellular EV distribution was analyzed using fluorescence microscopy (Agilent BioTek Cytation), with lysosomes labeled with LysoTracker Red. At each time point, cells were washed twice with PBS, followed by lysosome staining with 50 nM LysoTracker Red (Thermo Fisher Scientific, Waltham, USA) for 30 min at 37°C and nuclear counterstaining with DAPI for 15 min. Fluorescence signals were immediately visualized under the microscope.

### 4.13 Western Blotting

For cellular or tissue protein expression, Western blotting was performed as previously described [67]. In brief, total proteins were extracted by lysis of cells or tissues with RIPA lysis buffer (P0013B, Beyotime, China) containing a protease inhibitor mixture and then separated on a 10% SDS polyacrylamide gel and transferred to the polyvinylidene difluoride (PVDF) membrane (Millipore, Whatman). The PVDF membranes were blocked with 5% skim milk at room temperature (RT) for 2 h, and subsequently, the membranes were incubated with PTPRO (Cat. sc‐365354; Santa Cruz Biotechnology, CA, USA), PSD95 (Cat. No. 3409; Cell Signaling Technology, Beverly, MA, USA), synaptophysin (Cat. No. 5461; Cell Signaling Technology, Beverly, MA, USA), and β‐actin (Cat. No. 4970; Cell Signaling Technology, Beverly, MA, USA) at 4°C overnight. Then, the membranes were washed three times in Tris‐buffered saline with 0.1% Tween‐20 (TBST) solution for 10 min each and incubated with secondary antibodies for 2 h at RT. The protein bands were visualized with a high‐sensitivity chemiluminescence (ECL) kit (Bio‐Rad, USA).

### 4.14 Immunofluorescence Staining

Immunofluorescence was performed as previously described [68]. Briefly, 4 µm paraffin sections were cut from formalin‐fixed paraffin‐embedded specimens of mouse brain and were then deparaffinized and rehydrated, followed by endogenous peroxidase blocking and antigen retrieval. The following primary antibodies, DCX (Cat; sc‐271390; Santacruz), were incubated overnight at 4°C.

### 4.15 TUNEL Staining and Nissl Staining

As previously described, the ApopTag plus peroxidase in situ apoptosis detection kit was used to detect apoptotic cells. Nissl staining was performed according to the manufacturer's instructions (C0117, Beyotime Biotechnology). The stained sections were visualized under a microscope (Leica Microsystems), and the survival neurons and TUNEL‐positive cells in the hippocampal CA3 areas were counted using Fiji software.

### 4.16 In Vivo Imaging

Suspend EVs at a density of 1 g/L in DiR working solution. Incubate at 37°C for 20 min. Then it was centrifuged at 100 000 × g for 30 min at 4°C to remove the supernatant. Wash with PBS and centrifuge at 100 000 × g for 30 min to obtain pure DiR‐labeled exosomes resuspending 200 µL PBS. In the mouse model, EVs containing 150 µg of DiR‐stained EVs were injected into the tail vein of each mouse at 6 weeks of age [35]. Evaluation of exosome delivery in vivo by the IVIS Lumina imaging system began 3 h later. Images were acquired and analyzed with the use of Living Image version 4.4 software (Caliper LifeSciences). To determine the distribution of DiR‐labeled EVs in organs, images of organs (brains, livers, spleens, lungs, hearts, and kidneys) were collected and analyzed after the animals were euthanized. Adjust the freezing temperature regulator and freeze the tissue block on the freezer table. A short stay at room temperature was used for sectioning. The tissue block was trimmed, the required cut surface was exposed during sectioning, and the sections were attached to the slide after serial sectioning. The sections were fixed in 4% PFA for 30 min under dark operation and washed three times with PBS for 5 min each. Diluted DAPI was added and incubated for 20 min at room temperature. The separated organs were imaged by fluorescence microscopy.

### 4.17 Animal Experiments

CTRCI exhibits high prevalence in breast cancer, particularly among elderly patients [6]. DOX, one of the most effective chemotherapeutic agents for breast cancer treatment, has been shown to induce severe cognitive dysfunction in both clinical populations and animal models through multiple mechanisms [69, 70]. These include oxidative stress, neuroinflammation, DNA damage, impaired neurogenesis, synaptic dysfunction, apoptosis activation, and cell cycle disruption. Notably, the hippocampus appears to be particularly vulnerable to DOX‐induced CTRCI [70, 71]. Our previous work further identified age‐related decline in hippocampal PTPRO as a key mechanistic contributor to CTRCI. Collectively, in our study, we selected administration of DOX to establish mouse model of CRTCI to explore the effect of RVG‐EVs‐saRNA on aged CTRCI mice. The aged mice were randomly divided into the following groups (*n* = 10 per group): RVG‐EVs‐saCtrl/saline, RVG‐EVs‐sa*Ptpro*/saline, RVG‐EVs‐saCtrl/DOX, or RVG‐EVs‐sa*Ptpro*/DOX. DOX (2 mg/kg, Sigma–Aldrich) treatment dosage and schedule were established in previous studies [12]. For RVG‐EVs‐sa*Ptpro* treatment, the mice were treated with RVG‐EVs‐sa*Ptpro* (150 µg/mice) by tail vein injection one week earlier than DOX treatment for 5 continuous weeks [35]. At the end of the study, six mice from each group were collected to detect neuronal apoptosis and neurogenesis in the hippocampus. Three mice from each group were used for immunoblotting analysis.

### 4.18 Behavioral Analysis

Cognitive function in aged mice was assessed using the Y‐maze and Morris water maze (MWM) tasks, as previously described [12]. All behavioral tests were performed on mice aged 18–20 months old. Briefly, the spontaneous alternation percentage (SAP) in the Y‐maze task was recorded and evaluated as spatial working memory as described previously. The mouse was placed at the end of one arm of the apparatus as the start arm and was allowed to move freely in the Y‐maze during a 10‐min session. 3‐arm (each 30 cm long, 8 cm wide, and 15 cm in height) maze with equal angles between all arms. An alternation is defined as all three arms without reentry. The SAP is calculated as (the total number of alternations/the total possible alternations) ×100.

The MWM test consisted of two phases: the training phase and the probe phase. The experiment was conducted in a circular pool (150 cm diameter, 50 cm depth) filled with 24°C water, containing a hidden round platform (10 cm diameter) submerged 1 cm below the water surface. During the training phase, mice were placed in the pool from the west entry point and allowed a maximum of 60 s to locate the hidden platform, positioned in the northeastern quadrant. If a mouse found the platform within the allotted time, it remained there for 10 s. If the mouse failed to locate the platform within 60 s, it was gently guided onto the platform and allowed to stay for 10 s. The procedure was then repeated with the mouse introduced from the south entry point. Training sessions were conducted once daily for 5 consecutive days, with swimming velocity, escape latency, and total distance traveled recorded for each trial. On the sixth day, the probe test was performed by removing the platform and introducing the mice from the southwestern quadrant. Each mouse was allowed to swim freely for 60 s, during which the time spent in the target (northeastern) quadrant and the number of crossings over the former platform location were recorded. All behavioral data were acquired using the EthoVision 3.1 tracking system (Noldus Information Technology).

### 4.19 Golgi Staining

The FD Rapid GolgiStain kit (FD NeuroTechnologies) was used for Golgi‐Cox impregnation. The brains were removed and impregnated in Golgi‐Cox solution in the dark for 7 days. After polymerization, 150‐µm‐thick horizontal sections were cut using a vibratome and collected in a 0.3% gelatin solution. Finally, sections were dehydrated in alcohol and xylene and mounted on 0.3% gelatinized slides. Bright‐field images were acquired using a Cytation 5 multi‐mode plate reader (Agilent BioTek Cytation). Dendritic branches were traced, and their lengths were calculated using the Fiji plugin Simple Neurite Tracer. Sholl's analysis of proximal complexity was analyzed by NeuronStudio.

### 4.20 Hippocampal Slice Electrophysiology

As described previously [12], the mice were deeply anesthetized (Avertin, 13 µL/g, i.p.) and coronal sections (350 µm) were cut using a vibratome (VT1200S; Leica Microsystems) in ice‐cold artificial cerebrospinal fluid (ACSF, in mm: 119 NaCl, 2.5 KCl, 1 NaH_2_PO_4_, 11 glucose, 26.2 NaHCO_3_, 2.5 CaCl_2_, 1.3 MgCl_2_, and 290 mOsm, at pH 7.4. Recordings began following at least 30 min of incubation. Field excitatory postsynaptic potentials (fEPSPs) were recorded from the stratum radiatum of the CA3 region using a glass micro‐electrode (4–8 MΩ, filled with ACSF). Stimulation pulses (0.05 Hz) were delivered to the mossy fibers 100–150 µm away from the recording pipette using a bipolar tungsten stimulating electrode. The current intensity was adjusted to produce a fEPSP with an amplitude of 30%–40% of the maximal fEPSP response. After a stable baseline was established, LTP was induced with either one train or four trains of 1 s at 100 Hz after at least 20 min of stable baseline recordings. Data were collected and digitized by the MultiClamp 700 B (Axon Instruments). fEPSPs were recorded every 20 s and expressed as a percentage of average pretetanus baseline slope values.

### 4.21 Statistics

All analyses were conducted using Prism (version 7.0 b, GraphPad Software). Comparisons between two groups were performed with a student's *t* test or 1‐way ANOVA with post hoc intergroup comparisons, where appropriate. All statistical analyses of behavioral data were conducted using either 2‐way ANOVA or 3‐way ANOVA, where appropriate. All bar graphs show the mean ± SEM of at least three independent experiments. A *p* value of less than 0.05 was considered statistically significant.

## Author Contributions

H.Z. conceived and supervised the research. Z.Y., Y.M., H.L., and Y.J. performed most of the experiments and analyzed the data. Z.Y., Y.M., M.H., Q.L., and X.L. performed mouse behavioral experiments and associated data analysis. M.H. assisted with EVs preparation and animal experiments. H.R. and S.G. provided technical support for the preparation and characterization of EVs. X.Q., S.Z., Y.P., J.C., B.P., Z.G., and W.D. gave scientific advice and provided technical support. Z.Y., D.Z., L.Y., and H.Z. wrote the manuscript.

## Ethics Statement

The study was conducted in accordance with the principles of the Declaration of Helsinki. All the animal experiments were approved by the Animal Care and Use Committee of Jinan University (approval no. IACUC‐20240628‐07).

## Conflicts of Interest

The authors declare no conflicts of interest.

## Supporting information




**Supporting File 1**: advs76288‐sup‐0001‐SuppMat.docx.

**Supporting File 2**: advs76288‐sup‐0002‐DataFile.pdf.

## Data Availability

The data that support the findings of this study are available from the corresponding author upon reasonable request.
