# Supplementary material for: Mitigating Cancer Therapy–Related Cognitive Impairment by Targeted Activation of Undruggable Phosphatase
Source: Adv Sci (Weinh). 2026 Jul 28;13(52):e20135. doi: 10.1002/advs.202520135 (PMC13410807; doi:10.1002/advs.202520135)
Supplement: Supplementary file 2 [file ADVS-13-e20135-s001.docx]

**Mitigating Cancer Therapy–related Cognitive Impairment by Targeted Activation of Undruggable Phosphatase**

Zhimeng Yao^1,2†^, Yuhua Meng^2,3†^, Huanyi Li^4†^, Yinhui Jiang^2^, Xiaona Lin^2,5^, Mengyuan Hu^2^, Qing Liu^6^, Xiaofu Qiu^7^, Hongzheng Ren^8^, Yunlong Pan^9^, Bin Pan^1^, Zexiong Guo^1^, Shuyao Zhang^10^, Dianzheng Zhang^11^, Li Yang^12^, Shegan Gao^13*^, weijing Deng^4*^, Jianfan Chen ^1*^, Hao Zhang^14*#^

^1^Department of Urology Surgery, The First Affiliated Hospital of Jinan University, Jinan University, Guangzhou, Guangdong, China;

^2^State Key Laboratory of Bioactive Molecules and Druggability Assessment, MOE Key Laboratory of Tumor Molecular Biology, and Institute of Precision Cancer Medicine and Pathology, School of Medicine, Jinan University, Guangzhou, Guangdong, China;

^3^Postdoctoral Research Station of Basic Medicine, Jinan University, Guangzhou, Guangdong, China;

^4^Department of Pharmacy, The First Affiliated Hospital of Jinan University, Jinan University, Guangzhou, Guangdong, China;

^5^Department of Thoracic Surgery, the First Affiliated Hospital of Jinan University, Guangzhou, Guangdong, China;

^6^Department of Pathology, The First People’s Hospital of Foshan, Foshan, Guangdong, China;

^7^Department of Urology, Guangdong Second Provincial General Hospital, Guangzhou, Guangdong, China;

^8^Department of Pathology, Gongli Hospital, Naval Medical University, Shanghai, China;

^9^MOE Key Laboratory of Tumor Molecular Biology, and Department of General Surgery, The First Affiliated Hospital of Jinan University, Jinan University, Guangzhou, Guangdong, China;

^10^Department of Pharmacy, Jinan University Affiliated Guangzhou Red Cross Hospital, Guangzhou, Guangdong, China;

^11^Department of Biomedical Sciences, Philadelphia College of Osteopathic Medicine, Philadelphia, Pennsylvania, USA

^12^School of Life Sciences, Guangzhou University, Guangzhou, Guangdong, China;

^13^College of Clinical Medicine, The First Affiliated Hospital of Henan University of Science and Technology, Henan Key Laboratory of Cancer Epigenetics, Luoyang, Henan, China;

^14^School of Basic medical Sciences, Changzhi Medical College, Changzhi, Shanxi Province, China; Department of Pharmacy, Guangzhou Red Cross Hospital, Jinan University; State Key Laboratory of Bioactive Molecules and Druggability Assessment, MOE Key Laboratory of Tumor Molecular Biology and Institute of Precision Cancer Medicine and Pathology, School of Medicine, Guangzhou, China; Department of Pathology, Gongli Hospital of Shanghai Pudong New Area, Shanghai, China; Department of Thoracic Surgery, The First Affiliated Hospital of Jinan University, Guangzhou, China.

*Correspondence to: 1. haolabcancercenter@163.com; 2. doc_chen@yeah.net; 3. 427013885@139.com; 4. gsg112258@haust.edu.cn

**†**These authors contributed equally to the work.

#Hao Zhang is lead corresponding author.

**SI Figures and Figure legends**

**Supplementary Figure 1. Promoter-specific effect of the saRNAs on PTPRO expression.** (A) Schematic representation of *Ptpro* promoter showing TSS and target sites of four candidate saRNAs. (B-C) HT-22 cells were transfected with mock or candidate saRNAs at 30 nM concentration for 72 h. The mRNA expression level (B) and the protein level of PTPRO (C) were evaluated by RT-qPCR and immunoblotting. ***P* < 0.01, ****P* < 0.001 by one-way ANOVA followed by a Tukey-Kramer post hoc test.

**Supplementary Figure 2. Retention of Cy5 labeled saRNA after electroporation with EVs.** (A) The percentage of Cy5 labeled saRNA retained was measured by a fluorescent plate reader (excitation 560 nm and emission 610 nm) after 3 μg RVG-EVs mixed with 3 μg saRNA in 100 μL of buffer under different electroporation progress. (B) The percentage of Cy5 labeled saRNA retained was measured using the above method after 3 μg saRNA was electroporated into 3 μg RVG-EVs at 400V 125μF in the volume of buffer shown on the X-axis.

­­­­­­

**Supplementary Figure 3. The biodistribution of EVs in mice.** (A) Individual organs (i.e., liver, spleen, lung, heart, kidney, and brain) were isolated from mice treated with mock-EVs-saRNA or RVG-EVs-saRNA for 24 h. After tissue sectioning, representative images were acquired by confocal microscope. Red fluorescent spots indicate DiR-labeled EVs. Scale bar = 100 μm. (B­­­­) Colocalization of DiR-labeled mock EVs or RVG-EVs and RVG-HA tag in the mouse hippocampal CA3 region at 6h post-injection with mock EVs or RVG-EVs. Scale bar = 25 μm.

**Supplementary Figure 4. The persistence of *Ptpro* activation were evaluated in the hippocampus of the aged mice.** (A) Schematic illustration of saRNA treatment. (B-C) saRNA was administered to mice through the tail vein (n = 3 per group; 150 μg RVG-EVs-sa*Ptpro*-158 or mock-EVs-sa*Ptpro*-158, single dose). After 3, 7, 14, 21, and 28 days, the PTPRO expression in hippocampus was evaluated using by qRT-PCR (B) and immunoblotting (C). (D) No significant pathologic changes were found in the important organs of mice. Scale bar = 100 μm **P* < 0.05, ***P* < 0.01, ****P* < 0.001 by one-way ANOVA followed by a Tukey-Kramer post hoc test.

**Supplementary Figure 5. Representative IHC staining of PTPRO in the hippocampus.** PTPRO expression levels were significantly higher in the CA3 region compared to the CA1 and DG regions. Scale bars: 100 μm (upper panel), 50 μm (bottom panel).
