# Supplementary material for: Mitigating Cancer Therapy–Related Cognitive Impairment by Targeted Activation of Undruggable Phosphatase
Source: Adv Sci (Weinh). 2026 Jul 28:e20135. Online ahead of print. doi: 10.1002/advs.202520135 (PMC13410807; doi:10.1002/advs.202520135)

Original data of the corresponding images（advs.202520135）

Original data of the corresponding images in Figure 1

Figure 1B mock-EVs

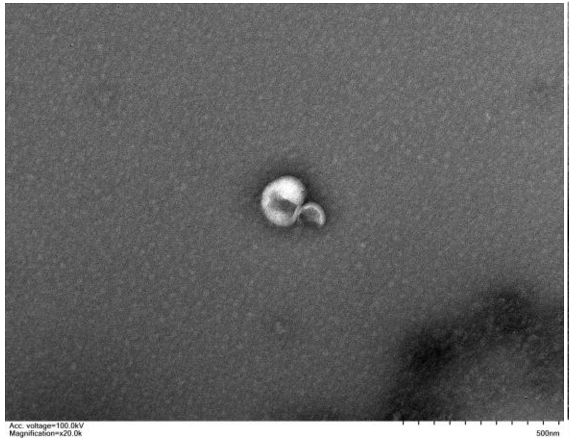

Figure 1B RVG-EVs

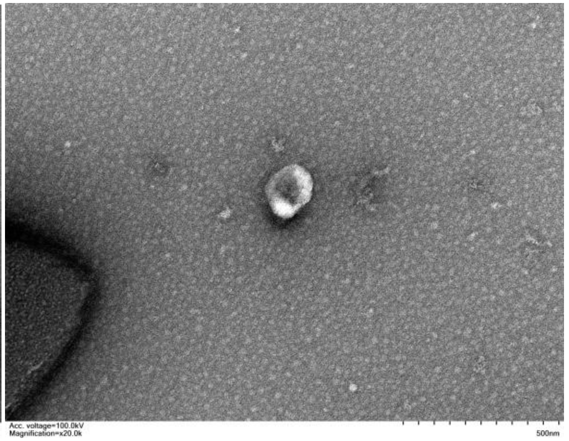

Figure 1B mock-EVs

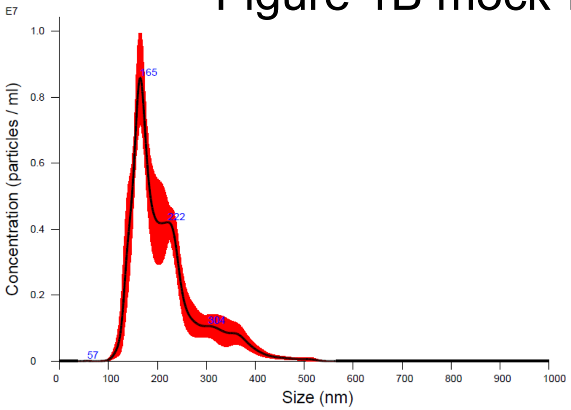

Figure 1B RVG-EVs

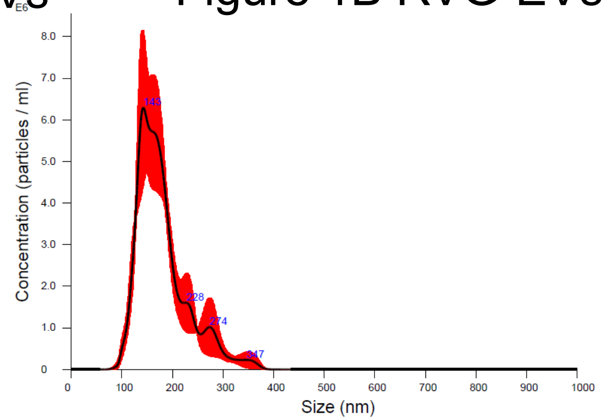

Figure1 D

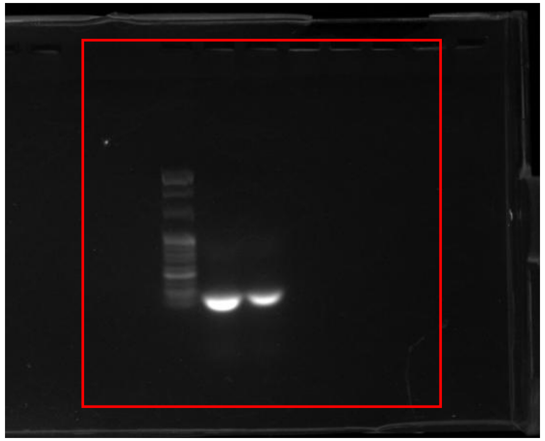

Full unedited gel for Figure 1C

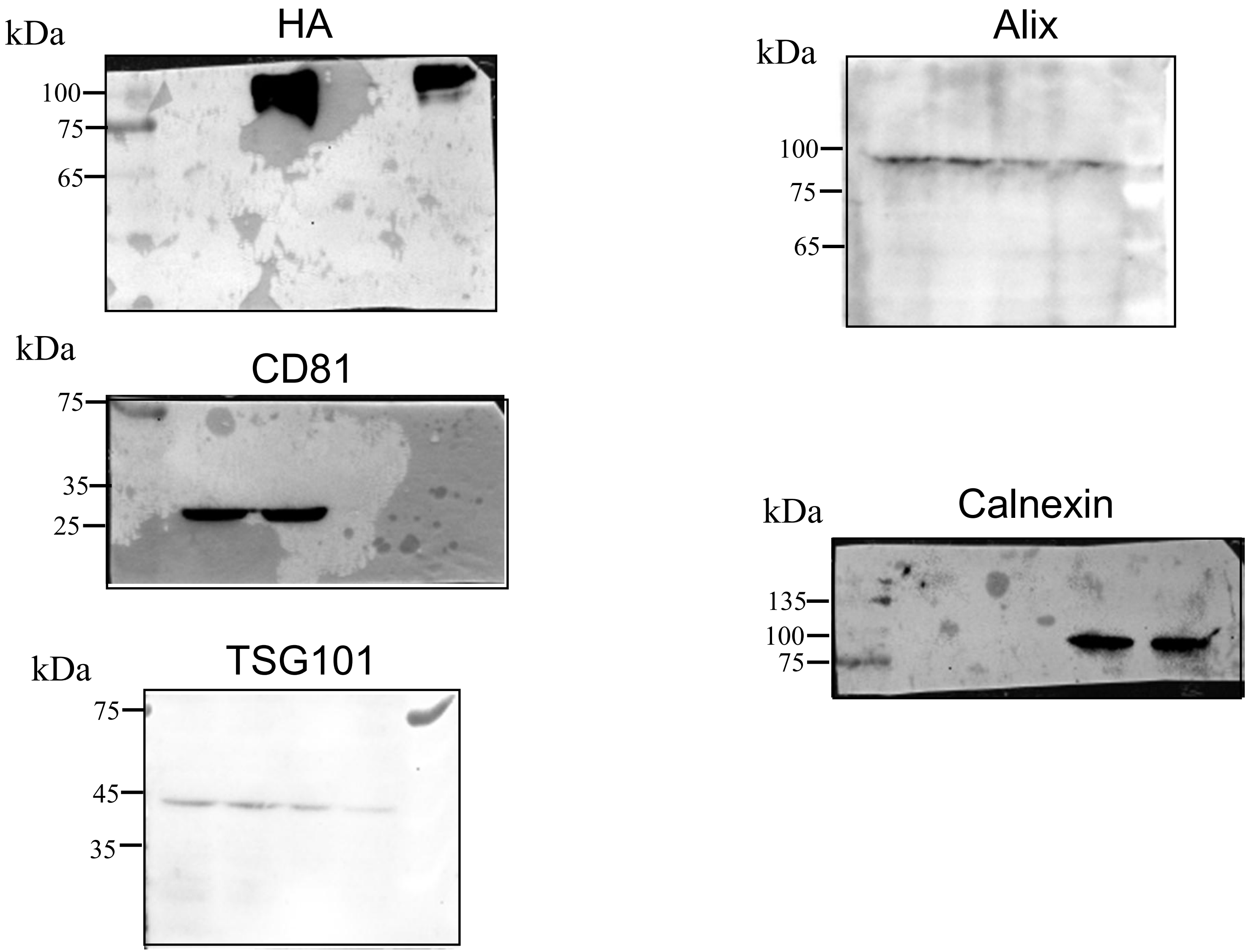

Original data of the corresponding images in Figure 2

Figure 2A

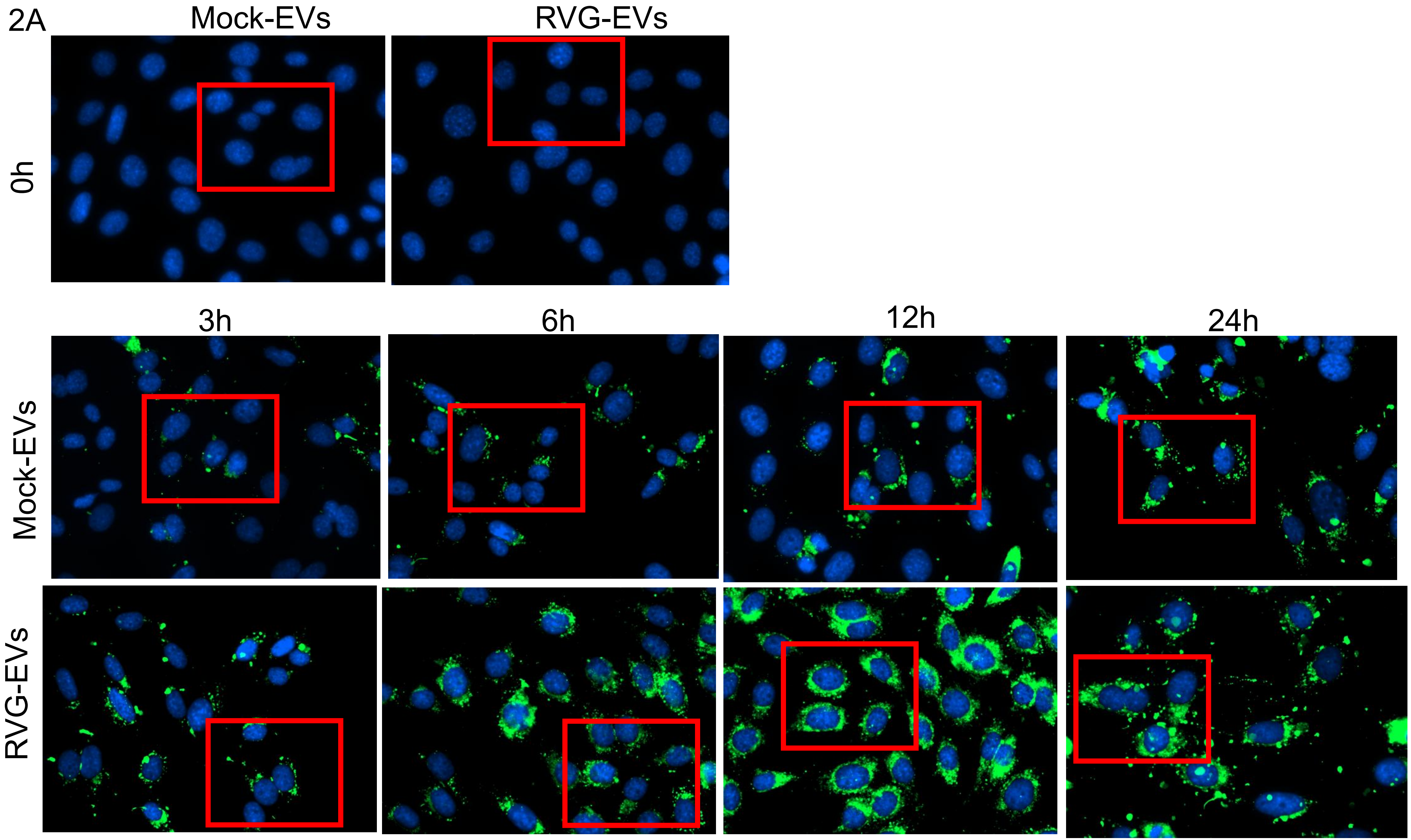

Original data of the corresponding images in Figure 2

Figure 2C

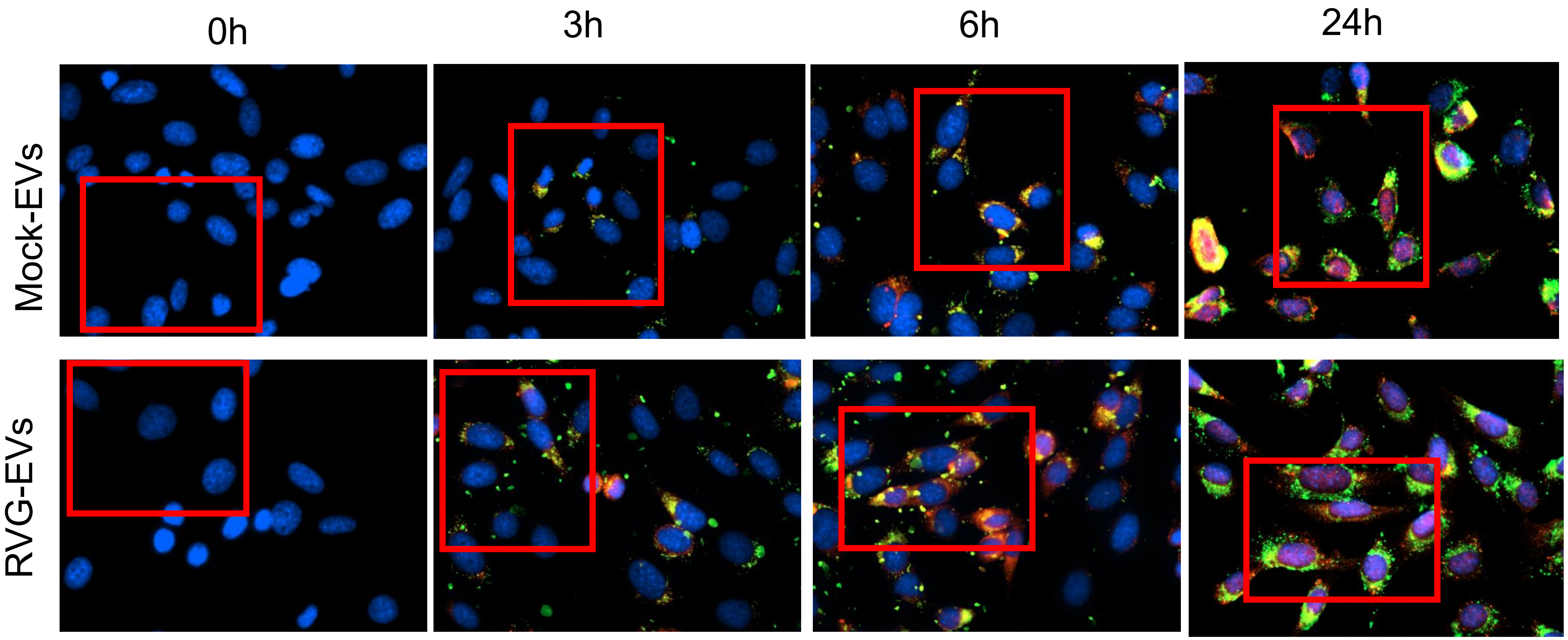

Original data of the corresponding images in Figure 2

Figure 2D

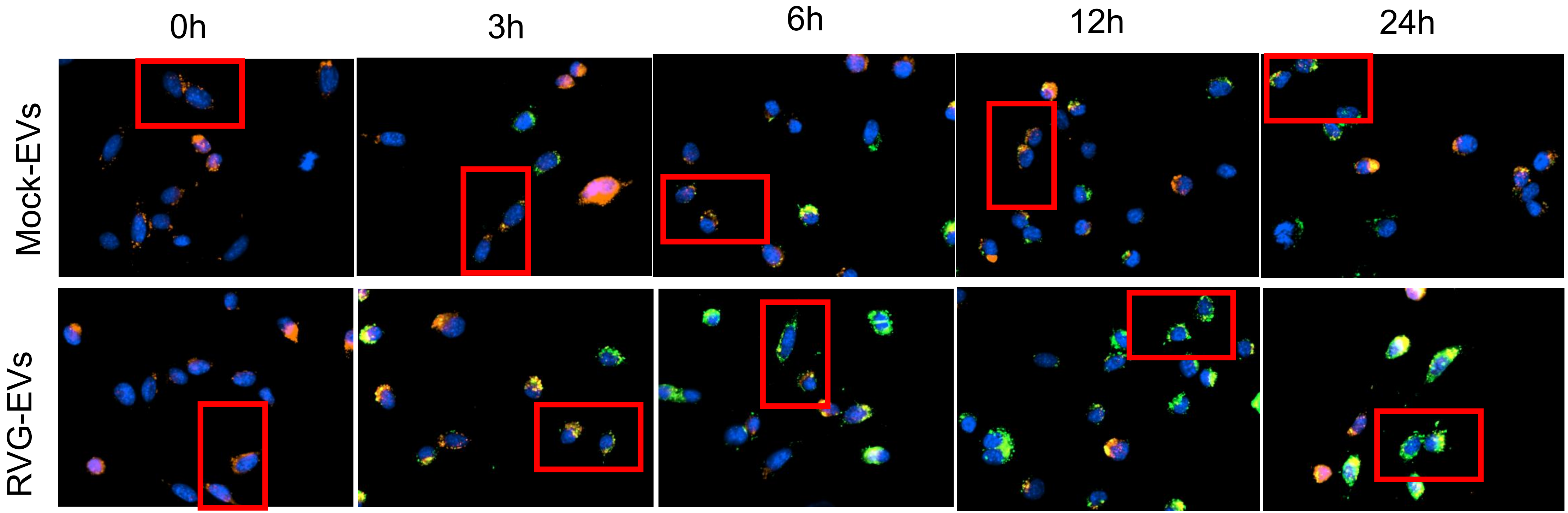

Full unedited gel for Figure 2E

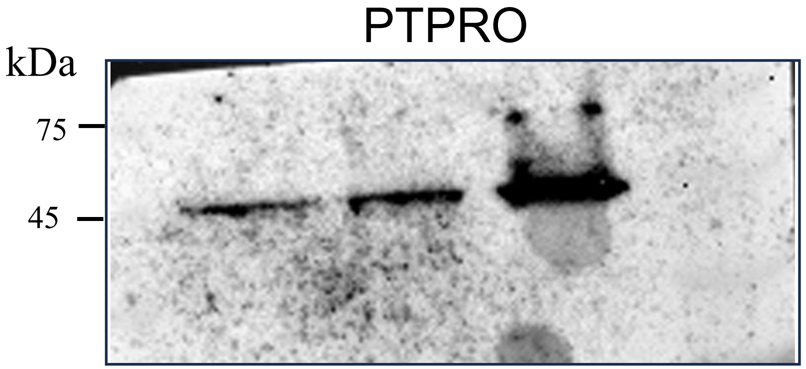

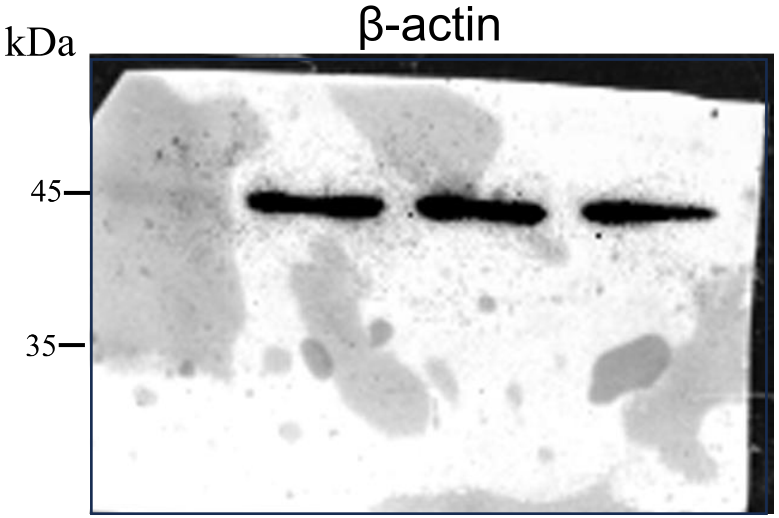

Original data of the corresponding images in Figure 3

Figure 3A

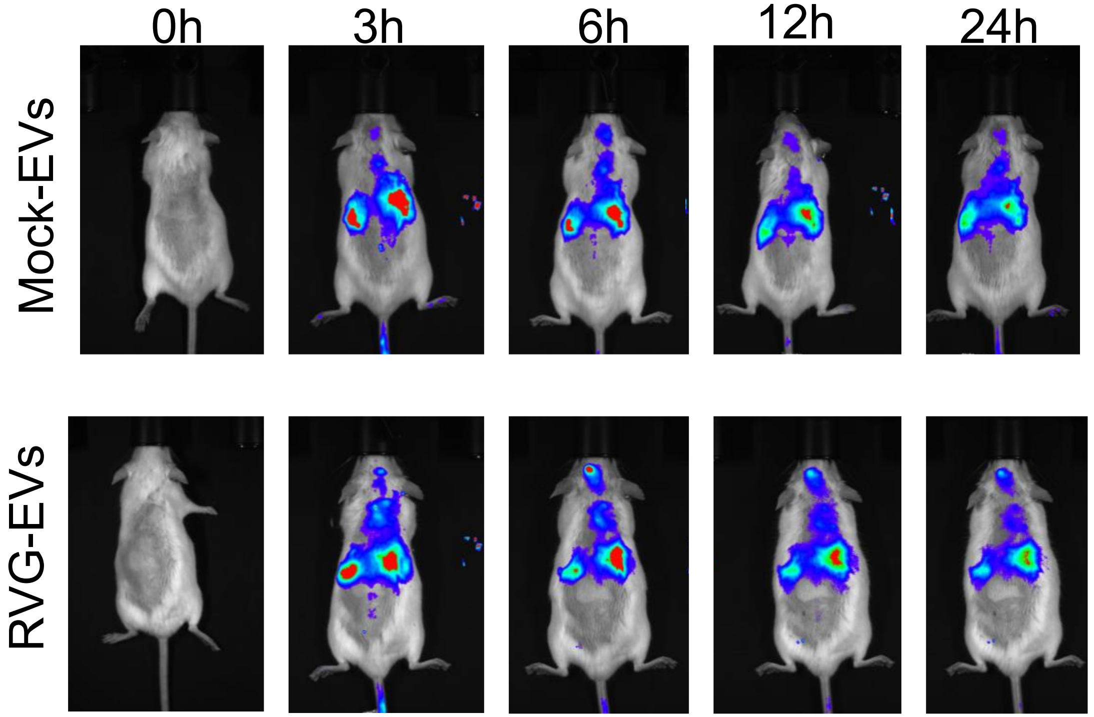

Figure 3B

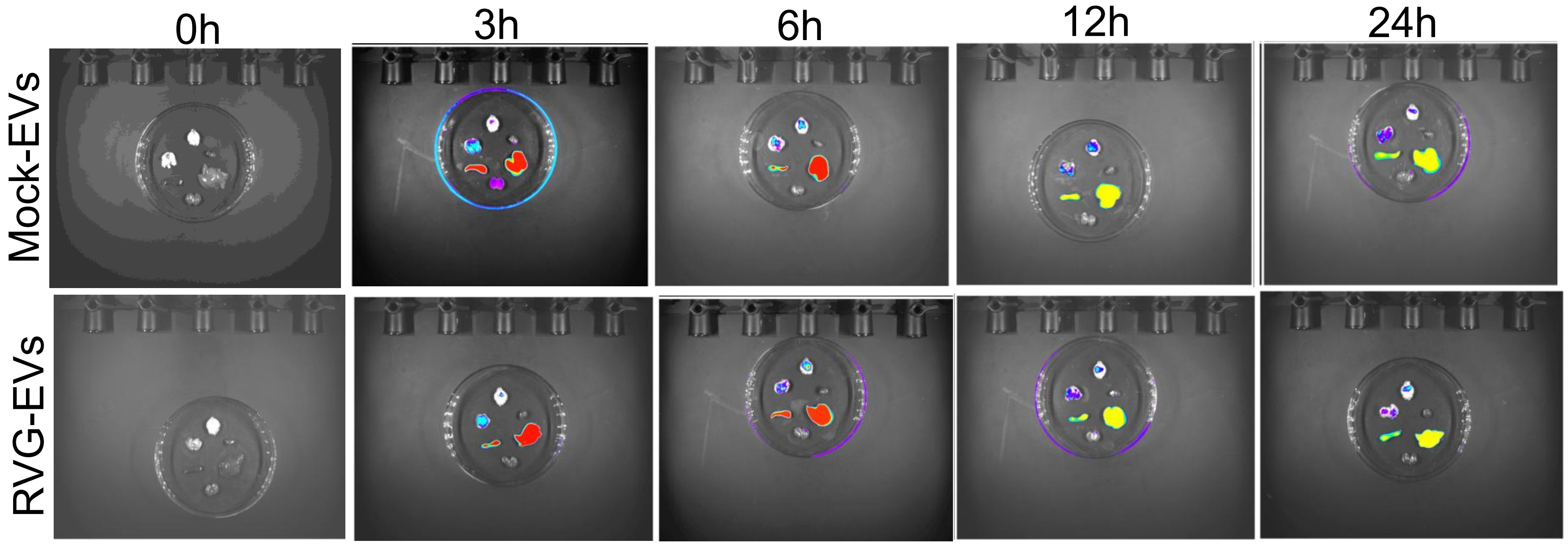

Original data of the corresponding images in Figure 3

Figure 3E

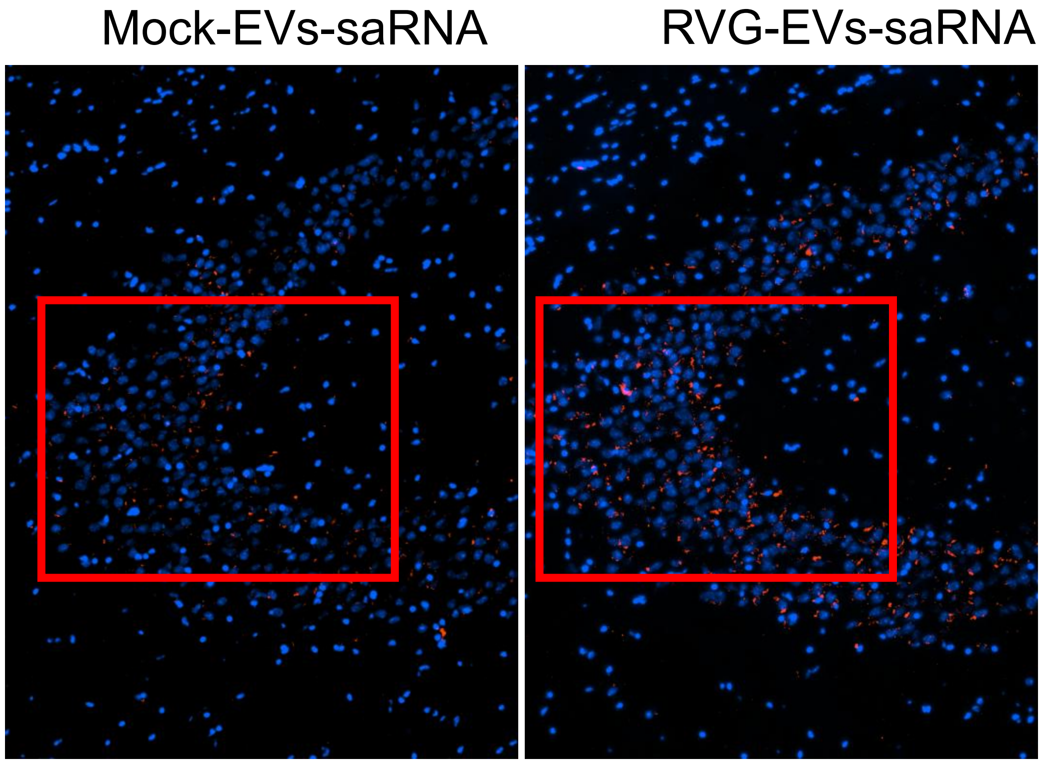

Full unedited gel for Figure 4C

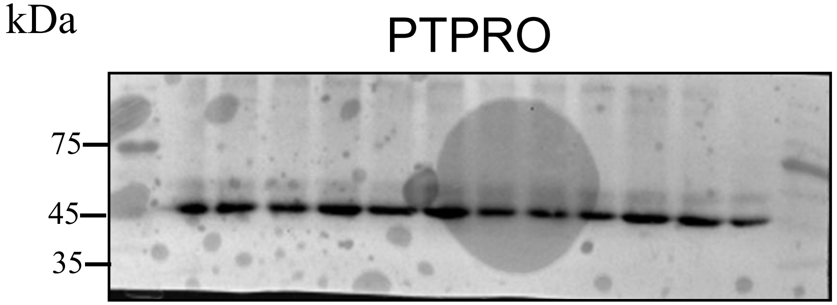

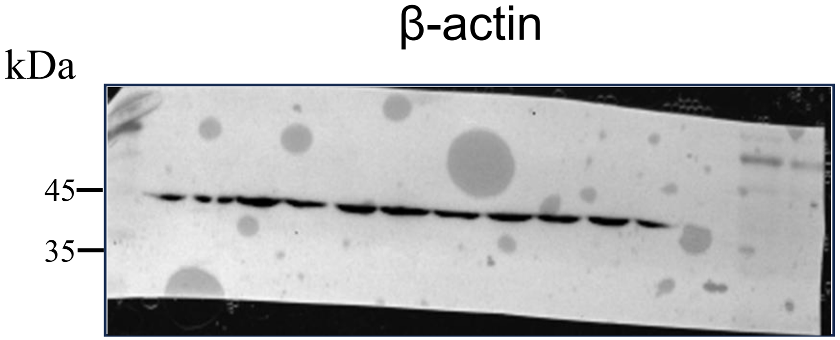

Original data of the corresponding images in Figure 4E

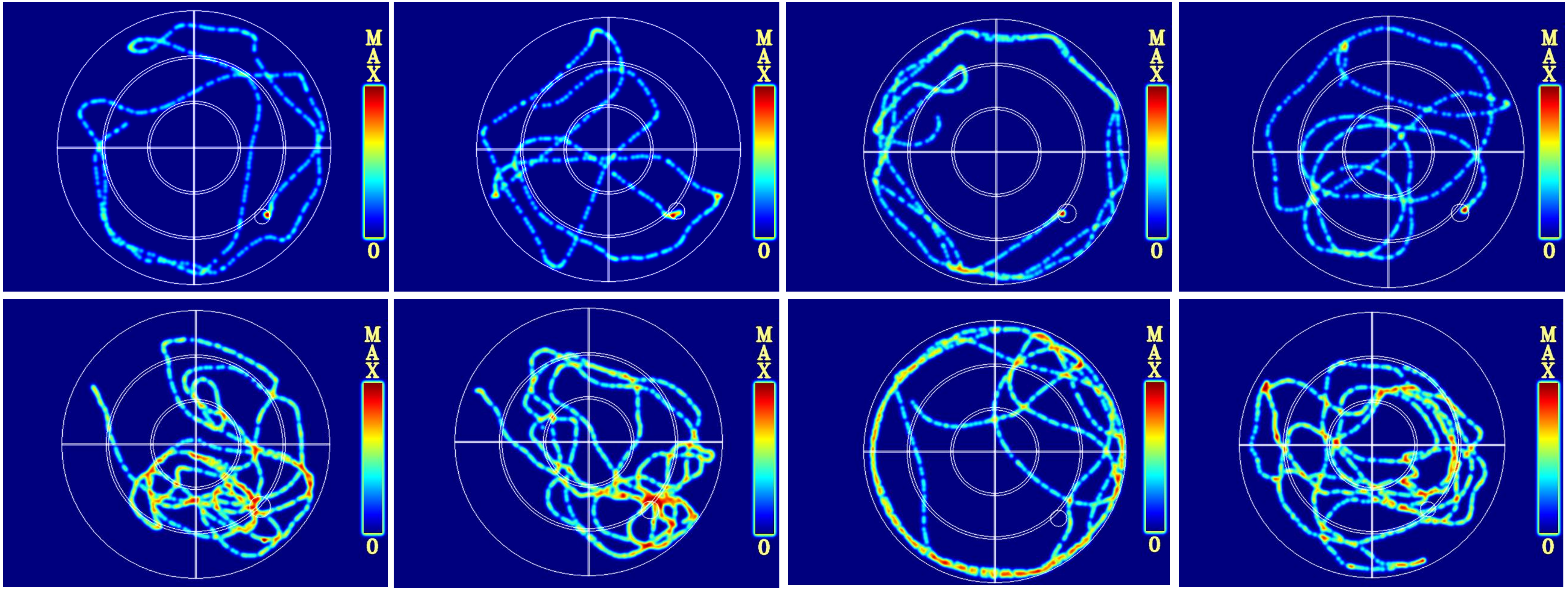

Original data of the corresponding images in Figure 5

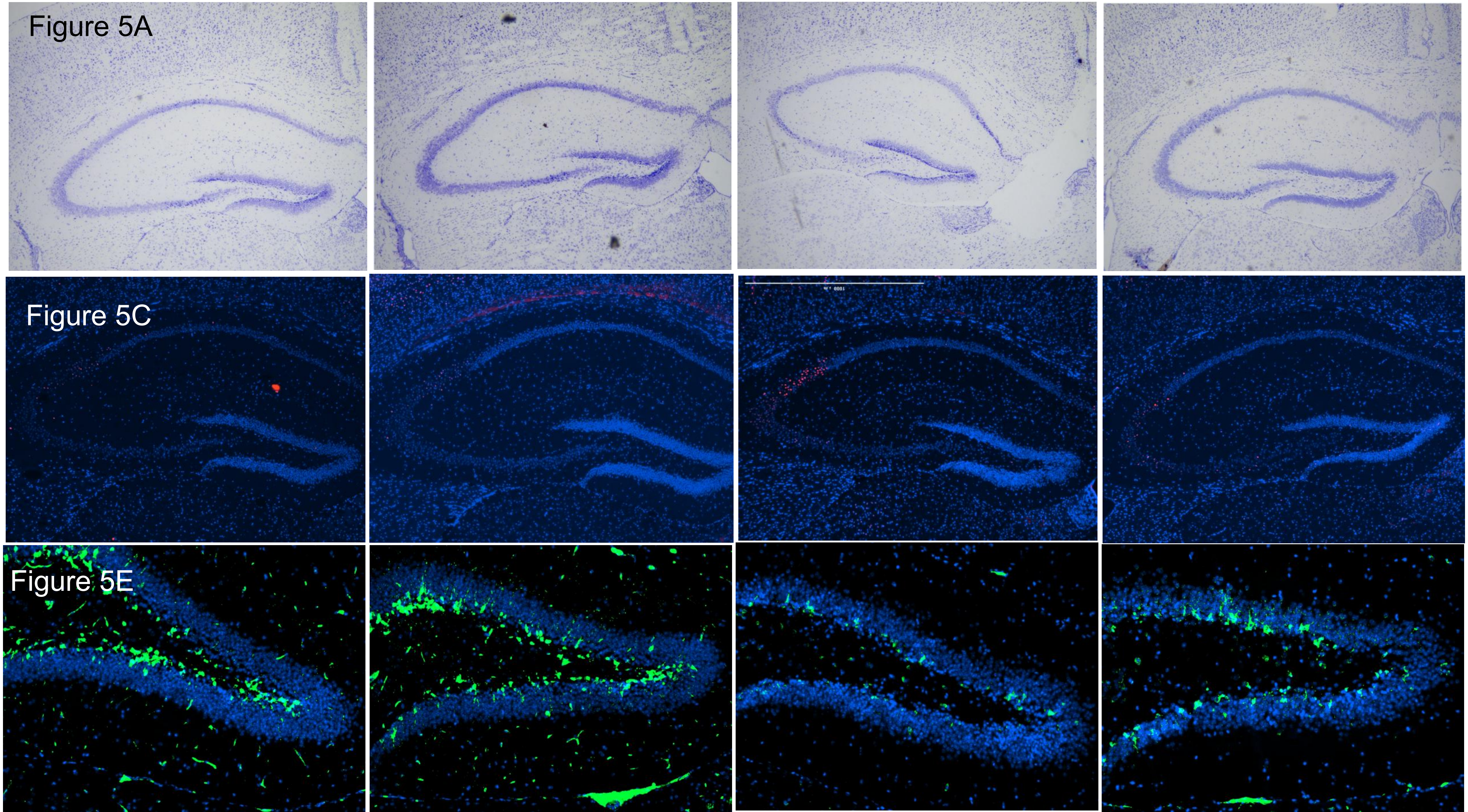

Original data of the corresponding images in Figure 6

Figure 6A

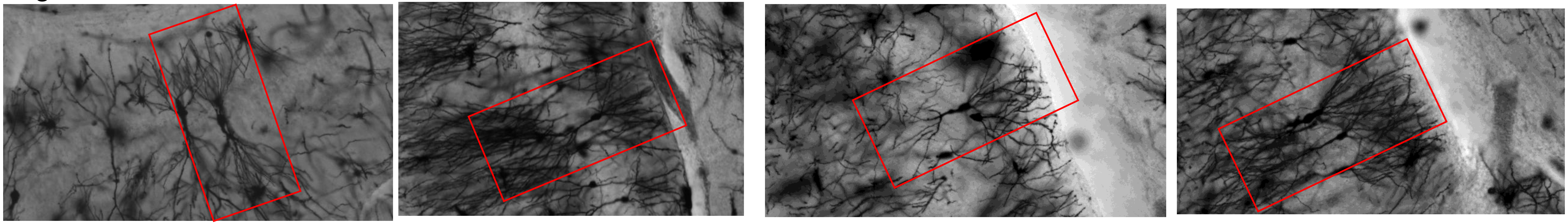

Figure 6E

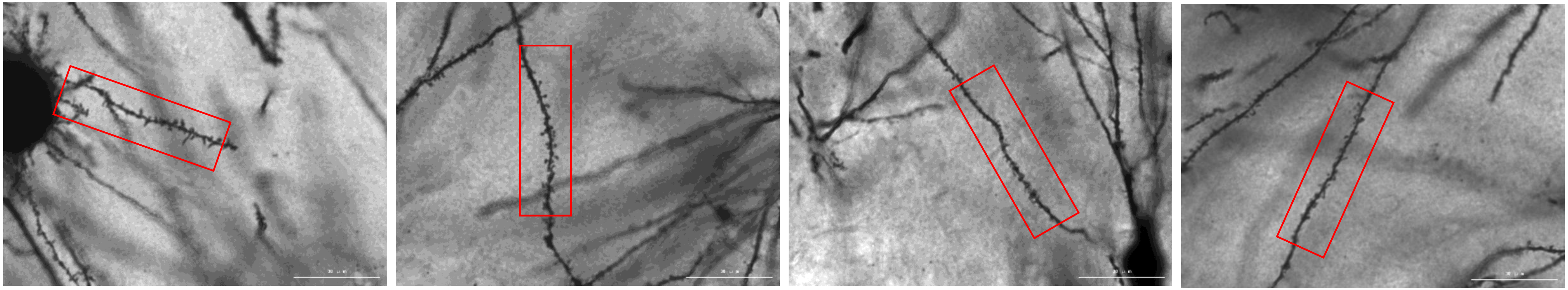

Original data of the corresponding images in Figure 6G

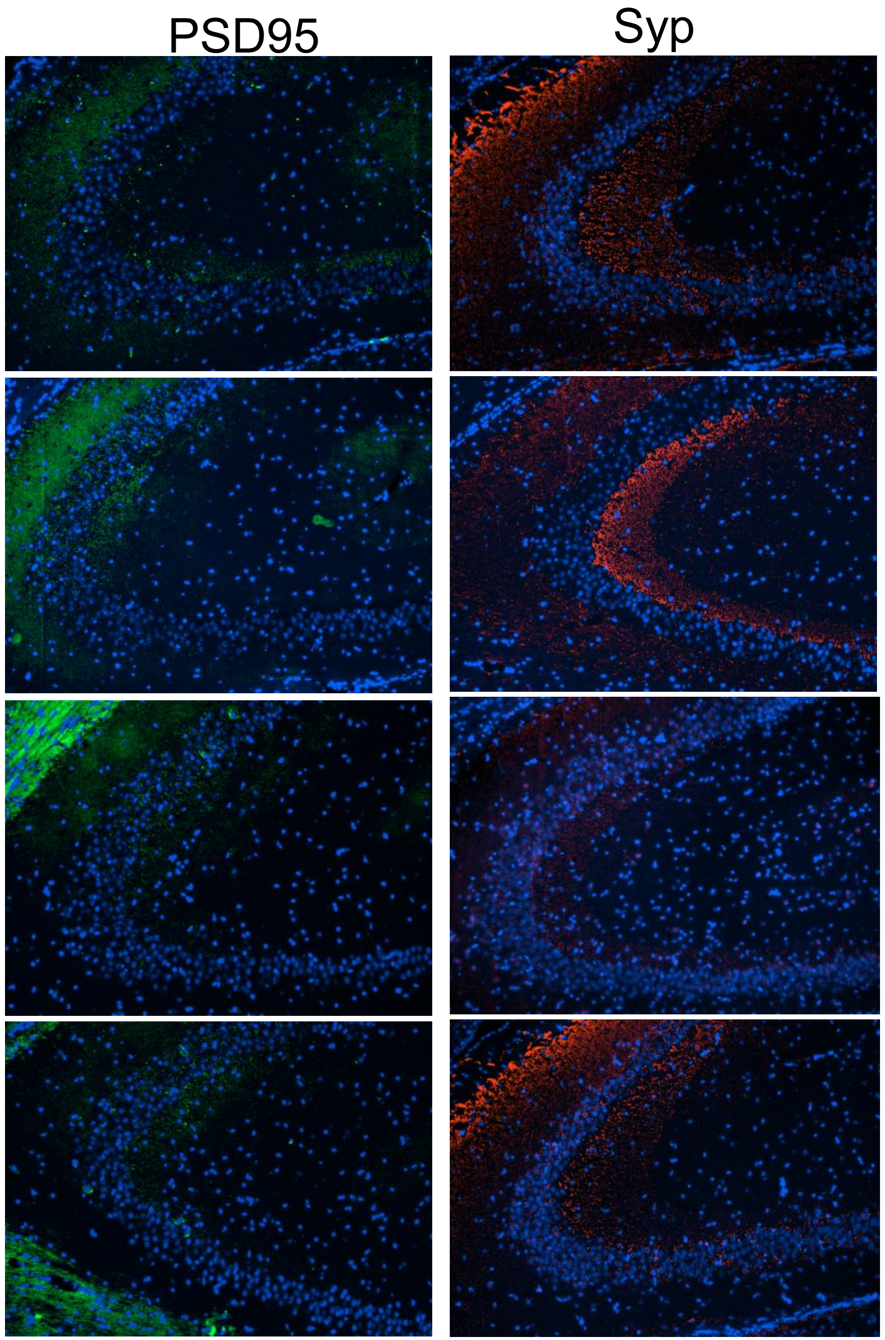

Full unedited gel for Figure 6J

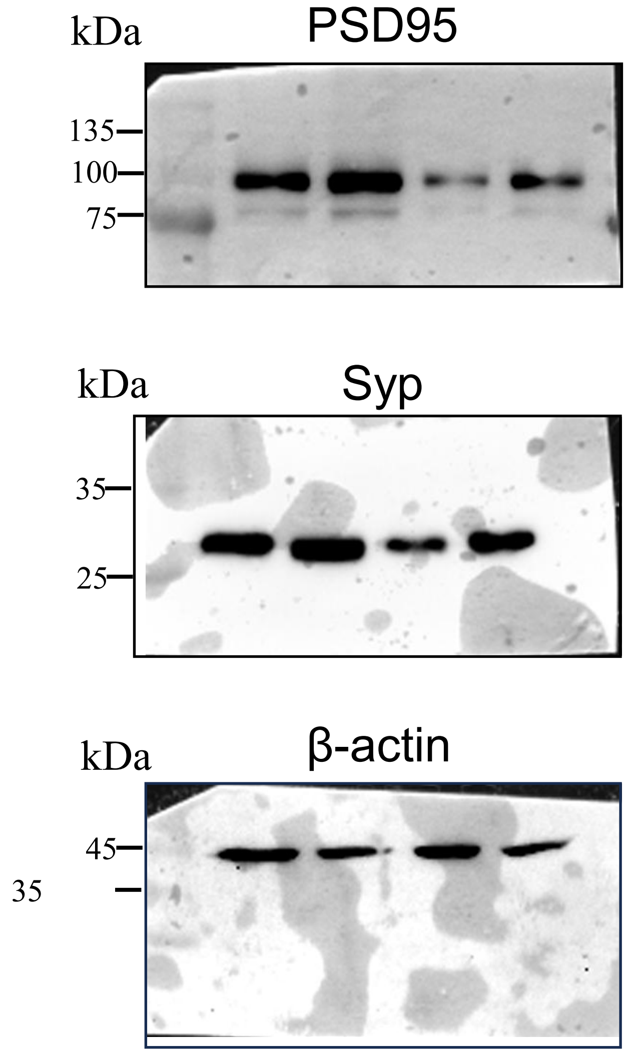

Full unedited gel for Supplementary Figure 1C

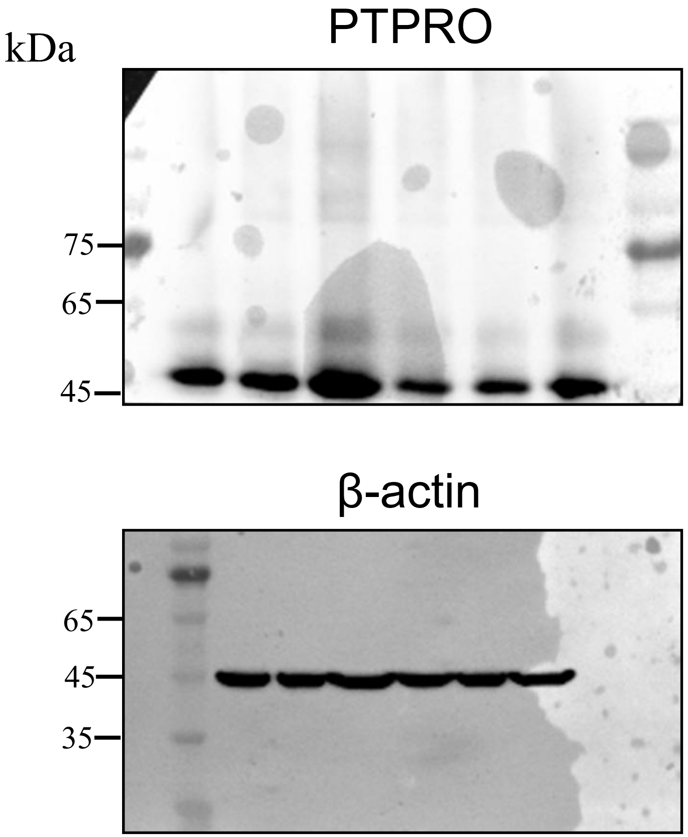

Original data of the corresponding images in Supplementary Figure 3A

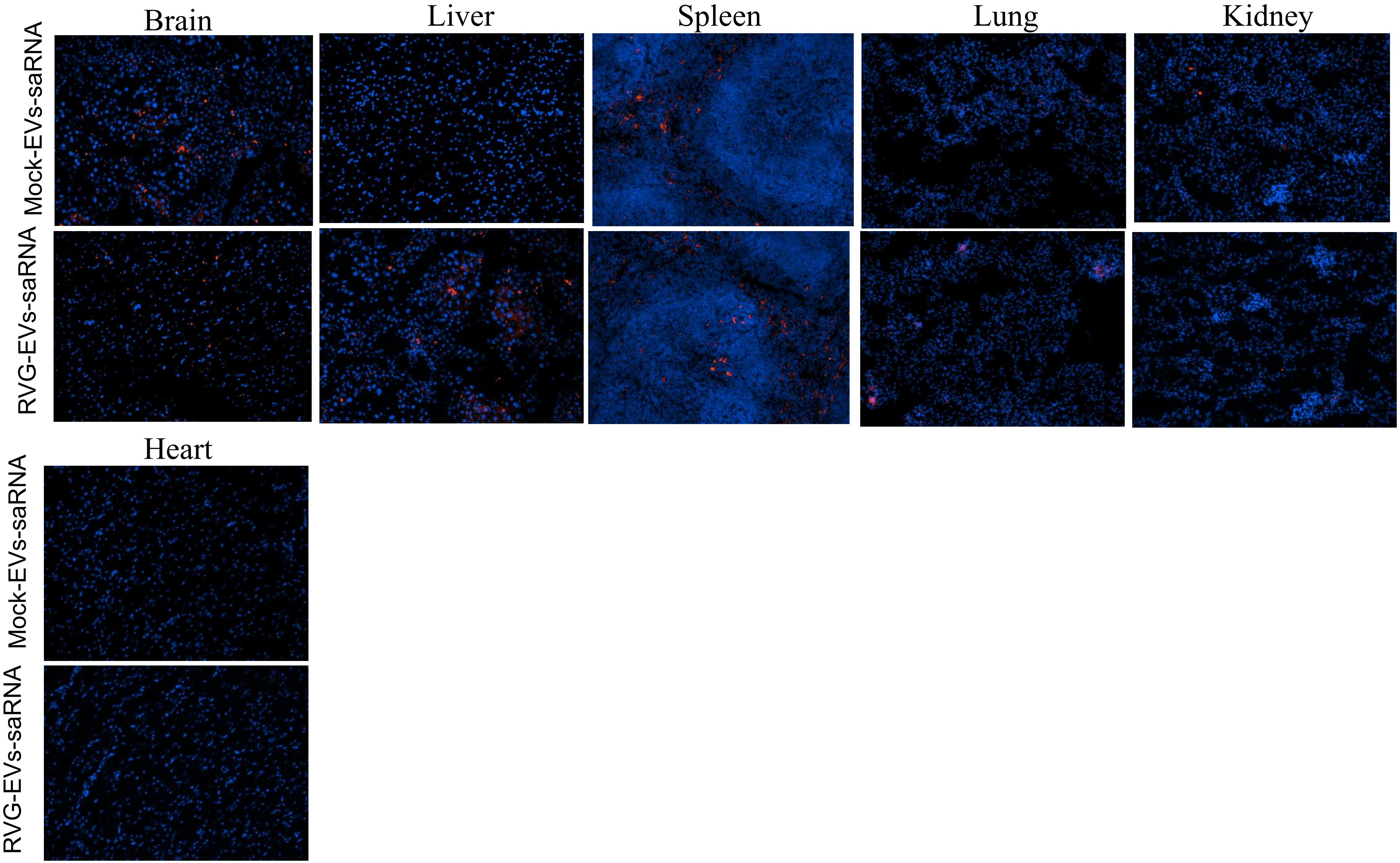

Original data of the corresponding images in Supplementary Figure 3B

Mock-EVs-saRNA

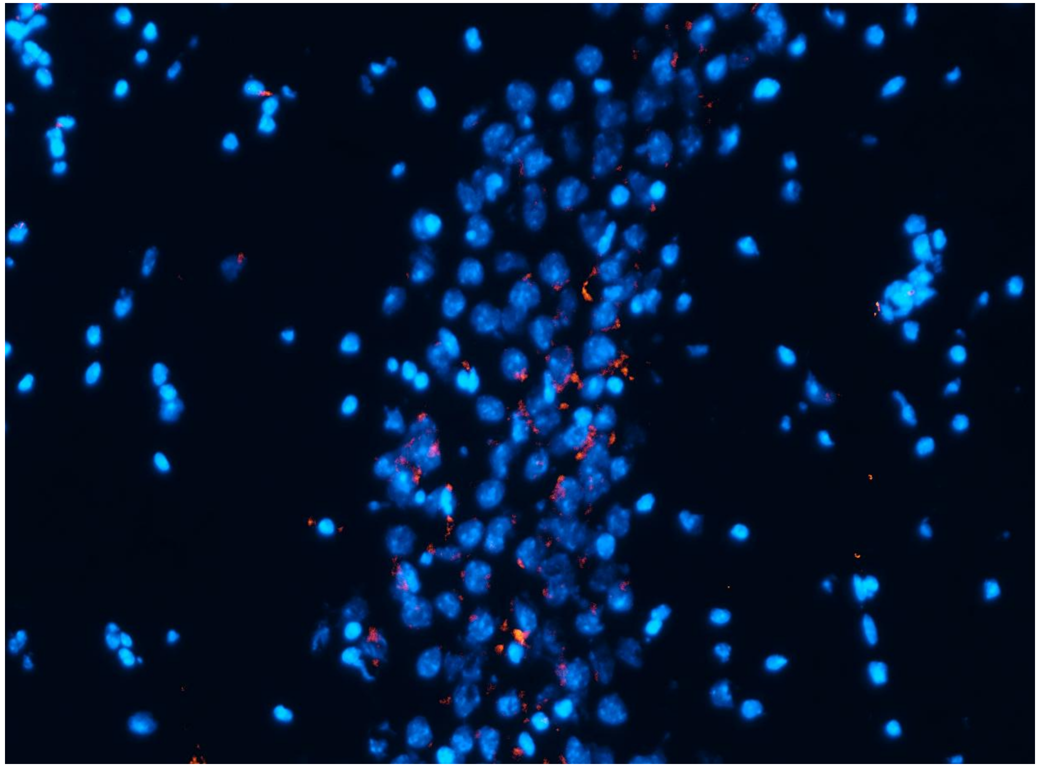

RVG-EVs-saRNA

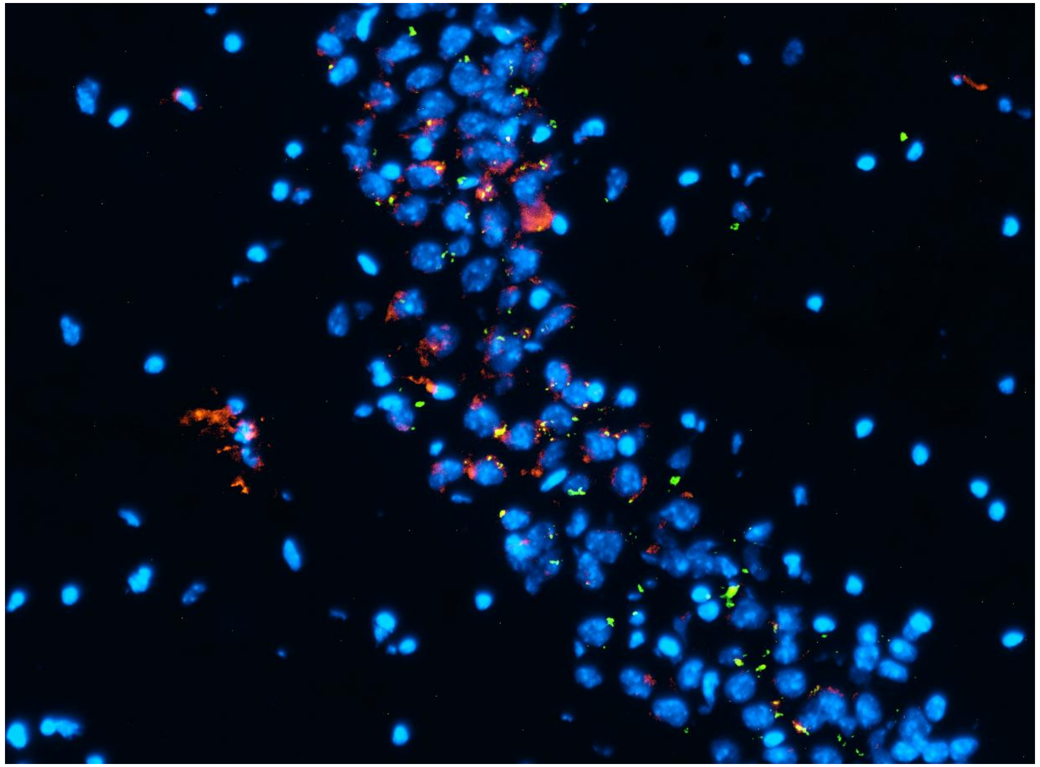

Full unedited gel for Supplementary Figure 4C

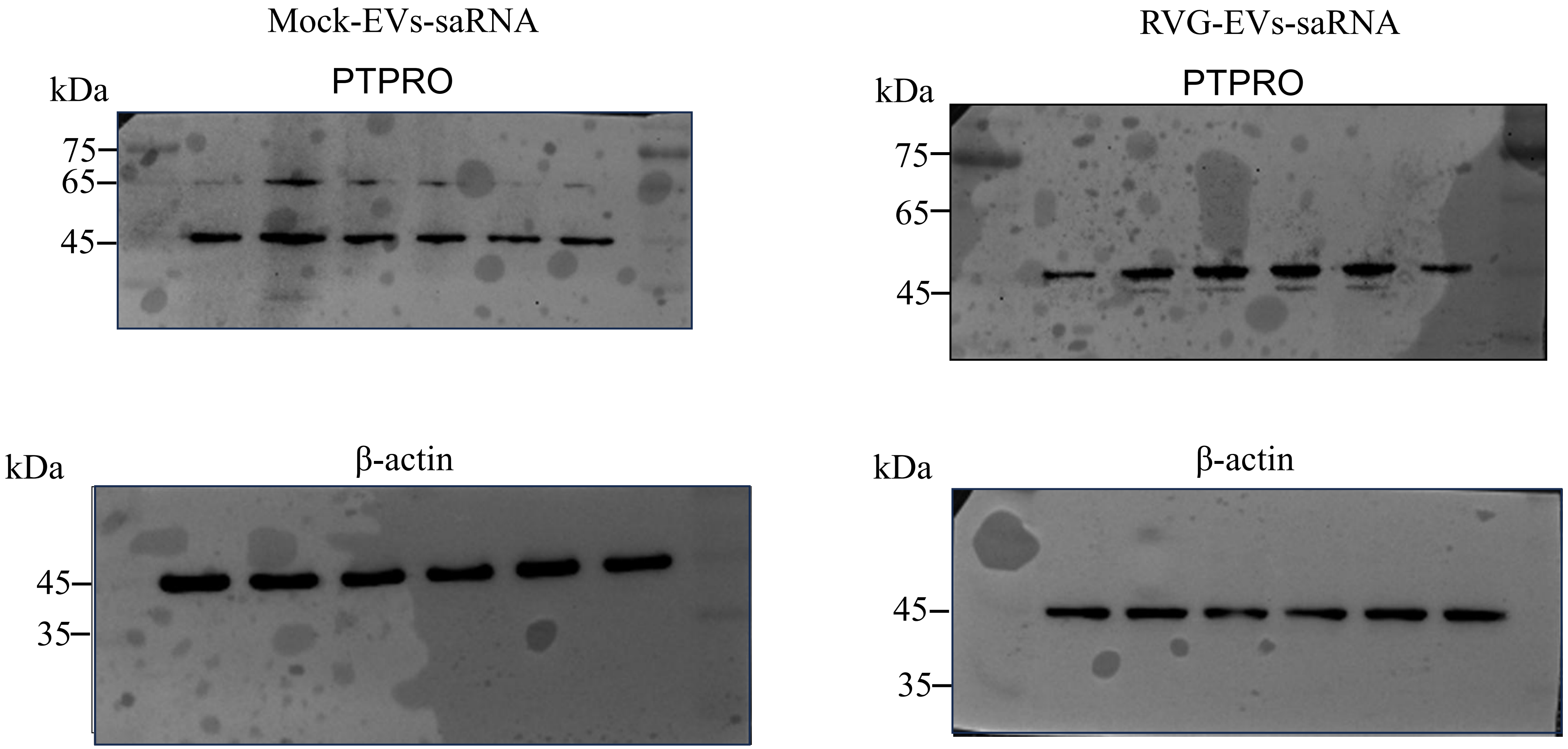

Original data of the corresponding images in Supplementary Figure 4D

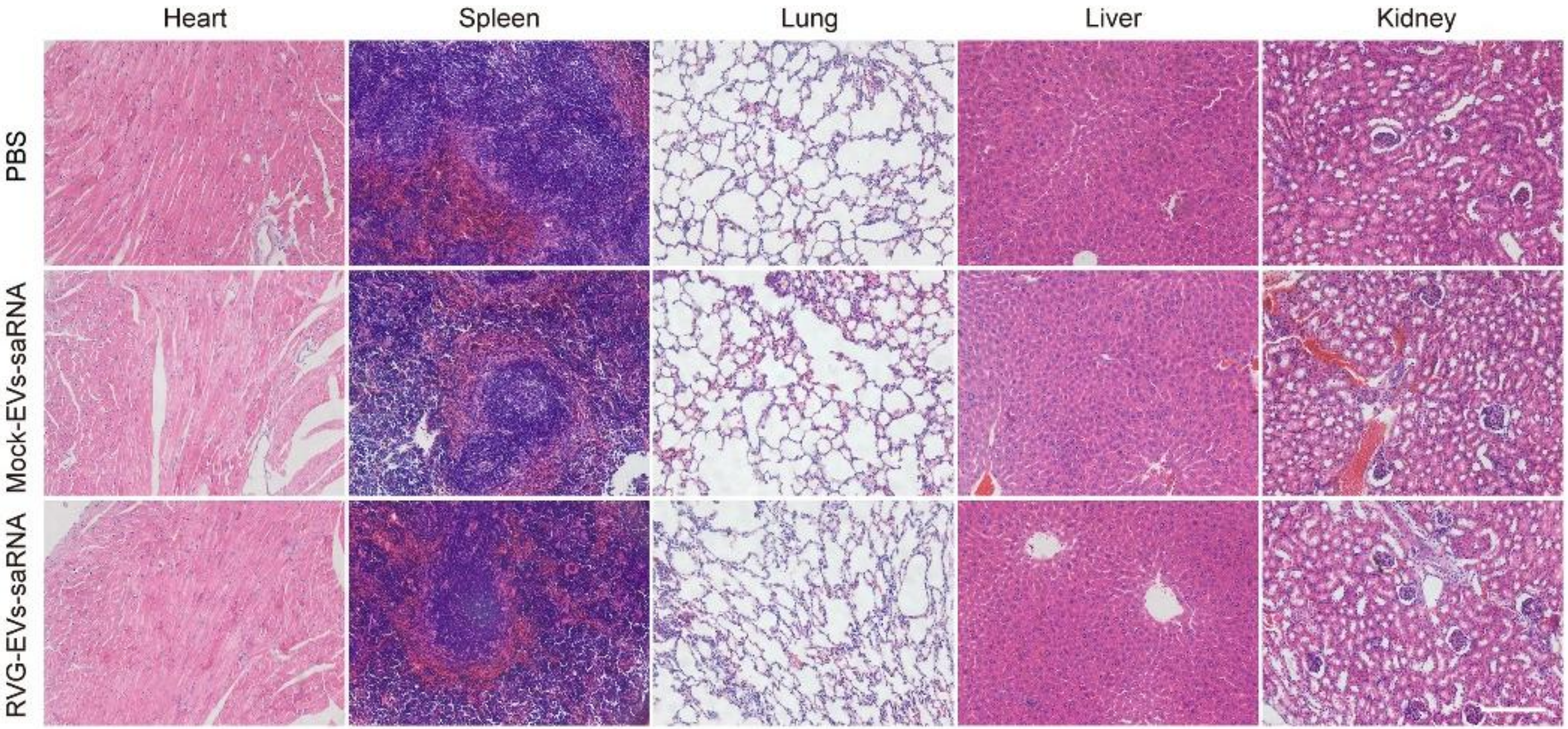

Original data of the corresponding images in Supplementary Figure 5

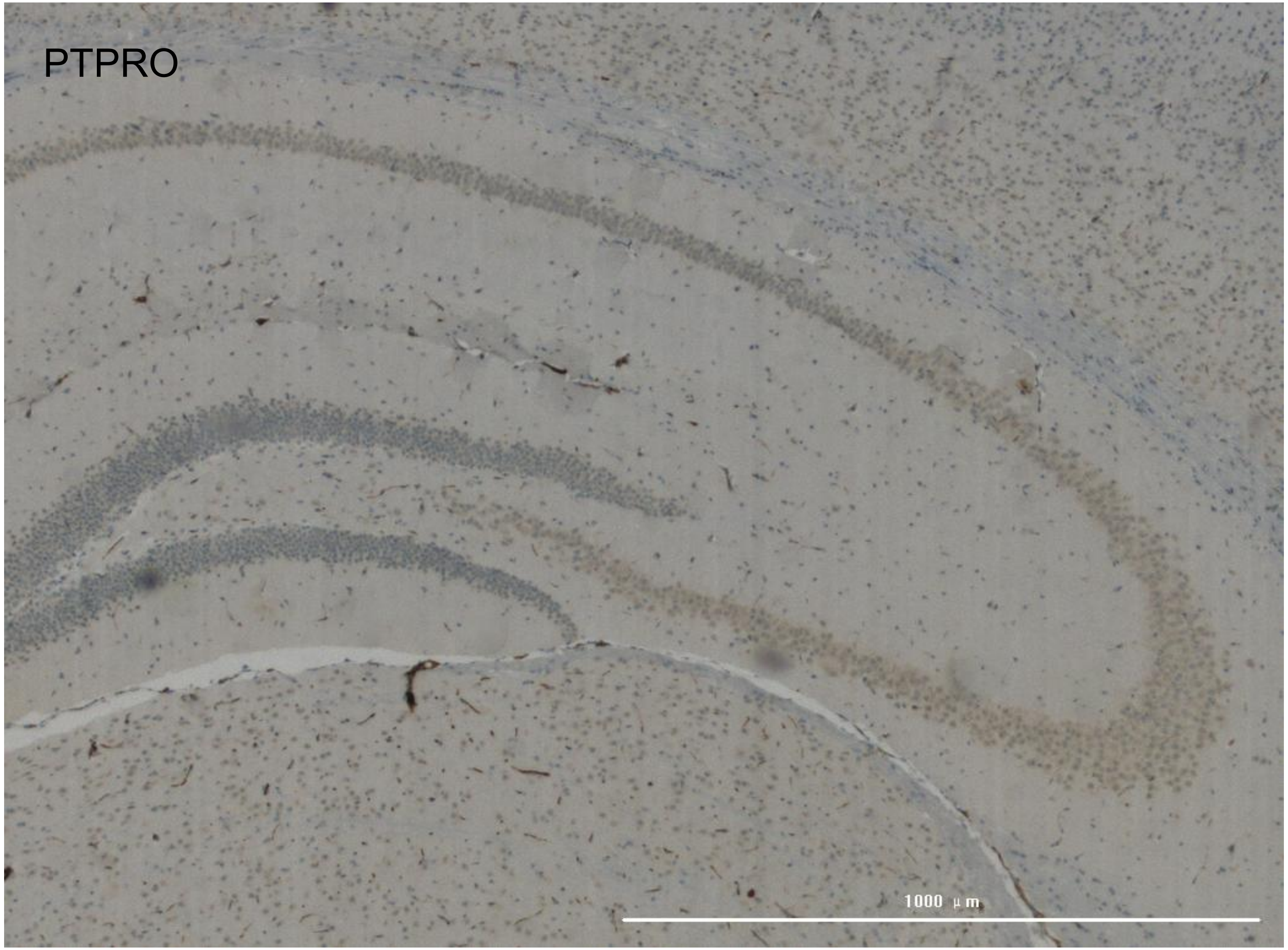

Supplement: Supplementary file 2 — Supporting File 2: advs76288‐sup‐0002‐DataFile.pdf. [file ADVS-9999-e20135-s001.pdf]
